# The Metabolic Regimes at the Scale of an Entire Stream Network Unveiled Through Sensor Data and Machine Learning

**DOI:** 10.1007/s10021-021-00618-8

**Published:** 2021-04-02

**Authors:** Pier Luigi Segatto, Tom J. Battin, Enrico Bertuzzo

**Affiliations:** 1 Stream Biofilm and Ecosystem Research Laboratory, Ecole Polytechinque Fédérale de Lausanne, CH-1015 Lausanne, Switzerland; 2 Department of Environmental Sciences, Informatics and Statistics, University of Venice Ca’ Foscari, 30170 Venice, Italy

**Keywords:** Stream metabolism, Streamwater temperature, Active radiation, River network, Network scale, Machine learning, Random Forest, Metabolic regime, Allochthonous respiration, Autochthonous respiration

## Abstract

Streams and rivers form dense networks that drain the terrestrial landscape and are relevant for biodiversity dynamics, ecosystem functioning, and transport and transformation of carbon. Yet, resolving in both space and time gross primary production (GPP), ecosystem respiration (ER) and net ecosystem production (NEP) at the scale of entire stream networks has been elusive so far. Here, combining Random Forest (RF) with time series of sensor data in 12 reach sites, we predicted annual regimes of GPP, ER, and NEP in 292 individual stream reaches and disclosed properties emerging from the network they form. We further predicted available light and thermal regimes for the entire network and expanded the library of stream metabolism predictors. We found that the annual network-scale metabolism was heterotrophic yet with a clear peak of autotrophy in spring. In agreement with the River Continuum Concept, small headwaters and larger downstream reaches contributed 16% and 60%, respectively, to the annual network-scale GPP. Our results suggest that ER rather than GPP drives the metabolic stability at the network scale, which is likely attributable to the buffering function of the streambed for ER, while GPP is more susceptible to flow-induced disturbance and fluctuations in light availability. Furthermore, we found large terrestrial subsidies fueling ER, pointing to an unexpectedly high network-scale level of heterotrophy, otherwise masked by simply considering reach-scale NEP estimations. Our machine learning approach sheds new light on the spatiotemporal dynamics of ecosystem metabolism at the network scale, which is a prerequisite to integrate aquatic and terrestrial carbon cycling at relevant scales.

**Supplementary Information:**

The online version contains supplementary material available at (10.1007/s10021-021-00618-8)

## Highlights

- Machine learning enables extrapolating stream metabolism, temperature, and light to the network scale.
- GPP, ER, and NEP change predictably across the river network.
- ER at network-scale allows for separation between allochthonous and autochthonous contributions.

## Introduction

Primary producers fix carbon dioxide (CO_2_) as organic carbon through photosynthesis, a flux known at the ecosystem level as gross primary production (GPP). Terrestrial GPP is the largest global carbon flux on Earth and drives critical ecosystem functions, foremost respiration, growth, and nutrient cycling. Terrestrial and marine GPP and ecosystem respiration (ER, as the respiration from all heterotrophic and autotrophic organisms) control land-atmosphere and ocean-atmosphere CO_2_ exchange with feedbacks on the world’s climate (Falkowski and others 1998; Heimann and Reichstein 2008; Beer and others 2010). The balance between GPP (positive flux) and ER (negative) flux is the net ecosystem production (NEP), which informs on the net accumulation of organic carbon and its potential trophic transfer, or on its export to other ecosystems. Understanding and predicting GPP and ER in terrestrial and marine ecosystems has been in a mainstay in global carbon cycling research and Earth system sciences (Chapin and others 2006; Goulden and others 2011; Xiao and others 2013).

Streams and rivers drain the continents in dense networks. Today we understand that the CO_2_ evasion fluxes from these ecosystems, including the smallest headwaters within the networks, are within the same range as ocean uptake fluxes of CO_2_, although in the opposite direction (Raymond and others 2013; Drake and others 2018; Horgby and others 2019b; Rocher-Ros and others 2019). Although these fluxes are becoming increasingly better constrained, the relative importance of CO_2_ sources within the catchment versus those from in-stream metabolism to the emitted CO_2_ is still often unclear (Hotchkiss and others 2015; Duvert and others 2018; Horgby and others 2019a). The proper quantification of metabolic fluxes in streams and rivers networks, and this across spatial and temporal scales, is therefore critical to pinpoint the role of these ecosystems in the global carbon cycle.

The advent of environmental sensor technology has profoundly changed the way we study stream ecosystem metabolism. Today, we are able to produce high-resolution time series of GPP, ER, and NEP at reach scale to draw conclusions on the drivers of stream ecosystem metabolic regimes (for example, Beaulieu and others 2013; Hall Jr and Beaulieu 2013; Hall Jr and others 2015; Bernhardt and others 2018). With such annual or even multiannual time series, the effects of shifts in the hydrological regime, including storm-induced disturbance and recovery dynamics, or of pulsed resource supply, for instance, can be studied (Uehlinger and Naegeli 1998; Raymond and others 2016; Demars 2019). Encompassing daily and seasonal variation of the metabolic regime, these time series are also critical to establish solid annual budgets of metabolic fluxes within streams, which are essential to assess catchment-scale carbon biogeochemistry and ecosystem fluxes (Tank and others 2018). In this context, NEP is particularly relevant as it links the terrestrial and aquatic carbon cycles (that is, the “boundless carbon cycle”, Battin and others 2009) through lateral carbon fluxes (Regnier and others 2013) and furthermore informs on the biogeochemical connectivity downstream and throughout fluvial networks (Battin and others 2008). Finally, understanding and predicting the metabolic regime of streams and rivers is also critical for ecosystem restoration at the nexus between hydrology, and both carbon and nutrient cycling (Palmer and Ruhi 2019).

Most studies on metabolic regimes refer to individual stream reaches (for example, Uehlinger and Naegeli 1998; Roberts and others 2007; Beaulieu and others 2013; Hall and others 2016; Appling and others 2018; Bernhardt and others 2018; Ulseth and others 2018), and therefore they do not allow to draw conclusions on potentially emerging properties at the scale of entire stream networks. This, however, would be essential to properly assess the relevance of streams and rivers for carbon cycling at regional and global scales. One noticeable exception is the study by Rodriguez-Castillo and others (Rodríguez-Castillo and others 2019) using spatial statistics to extrapolate ecosystem metabolism from 41 reaches over 72 hours to a stream network. While this study helps to resolve the spatial variation of stream ecosystem metabolism within a network, it does not inform on its temporal dynamics. The combination of both spatial and temporal variation (that is, the regime) of stream metabolism at the level of entire networks has been hampered by the lack of suitable modeling approaches. Resolving this issue is relevant for the proper upscaling of metabolic fluxes in time and space and to detect emerging properties of stream networks. Owing to the dendritic and other topological characteristics of stream networks, the hydrological regime and its resilience are not uniformly distributed across a given network (Botter and others 2013). Furthermore, network structure seems to affect the community structure of stream biofilms (Widder and others 2014), which orchestrate numerous ecosystem processes, including metabolism (Battin and others 2016). A recent series of theoretical studies using optimal channel networks have highlighted the potential role of stream network emerging properties for the removal of dissolved organic carbon and nitrogen, and for GPP regimes (Bertuzzo and others 2017; Helton and others 2018; Koenig and others 2019).

Machine learning is now offering novel opportunities to advance geosciences at the interface between climate and global carbon models (for example, Reichstein and others 2019), for instance, but also ecological sciences (for example, Olden and others 2008). Despite the power of machine learning to resolve complex nonlinear or multimodal relationships as they often underlie Earth’s surface and ecological processes, to date, it remains poorly used in stream biogeochemistry. Here, we propose that machine learning has the potential to extend stream ecosystem metabolism from the reach scale to the scale of entire stream networks. Over the last years, a suite of environmental predictors for stream ecosystem GPP and ER has been identified (for example, Mulholland and others 2001; Beaulieu and others 2013; Bernhardt and others 2018). Chief among them figure photosynthetic active radiation (PAR) and temperature (T). Machine learning could thus be used to extrapolate both in time and space such heterogeneous forcings (for example, streamwater temperature and light) required, for instance, to run process-based models for reach-scale metabolism (Hall and others 2016; Segatto and others 2020) to the scale of an entire stream network. Furthermore, the same procedure could be tested directly on reach-scale estimates of ecosystem metabolism to check whether available data contain enough information to explain network-scale variations in metabolic regimes.

**Figure 1:**
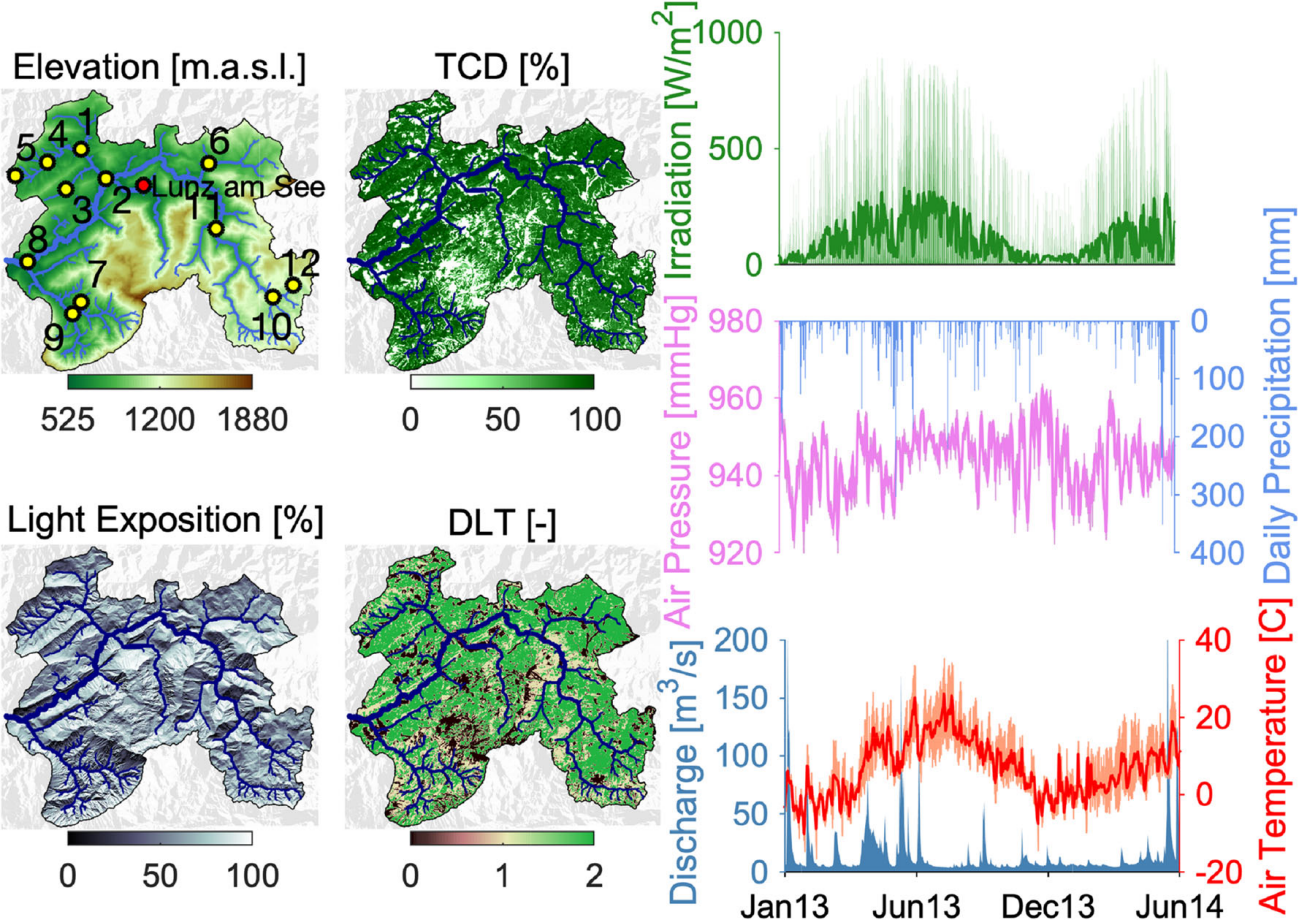
Ybbs Catchment and related major spatiotemporal covariates employed in the learning process: elevation, tree cover density (TCD), dominant leaf type (DLT), and light exposition (top four panels, clockwise order). Sampled reaches (yellow dots numbered from 1 to 12) and the weather station (red dot) are shown as well. Major temporal covariates are displayed from January 2013 to June 2014 in the right-hand side of figure. High-frequency measurements of irradiation, air pressure, precipitation, and air temperature have been collected at the Lunz am See weather station. Hourly discharge displayed in the last subplot refers to the measured signal at the outlet of the catchment. Light and air temperature are shown both at hourly (thin line) and daily (thick line) timescale.

In this study, we used Random Forests (RF, Breiman 2001) as a supervised learning technique to describe and predict patterns of metabolic regimes (that is GPP and ER) and critical environmental forcings (that is PAR and T) at the scale of an entire stream network. To do so, we used 18-month-long time series of data available for the Ybbs River network (Austria, Figure 1). Streamwater temperature, photosynthetic active radiation, and dissolved oxygen concentration were available for all 12 reach sites (Ulseth and others 2018). Furthermore, we used such data to estimate daily rates of ecosystem GPP, ER, and NEP via the single-station approach (see “Methods” section). We explicitly trained our RF model by integrating distal factors, such as vegetation type, canopy cover, hydraulic flow geometry, hydrogeomorphic properties, incident light, precipitation, and other climatic variables.

This approach allowed us to reliably establish annual regimes of water temperature, light, and metabolic fluxes (GPP, ER, and NEP) across the Ybbs River network and to quantify the relative contributions from small and large streams to these fluxes at the network scale. We were also able to quantify the annual variability of each of these fluxes across the network with the goal to assess the metabolic resilience from small to larger streams. Finally, we partitioned the allochthonous and autochthonous components of ER, which is important to assess allochthony at the network level. Our findings shed new light on stream network metabolic regimes and provide empirical evidence for long-standing theory predicting shifts of ecosystem metabolism along the stream continuum (Vannote and others 1980).

## Methods

### Study Area

We combined our machine learning approach with data from the Ybbs River network, which drains a 256 km^2^ prealpine catchment in Austria (47°48’22.9” N, 14°57’00.8° E). The stream network is shown in Figure 1 and has been delineated from a 10-m-resolution digital elevation model (DEM) (Besemer and others 2013). The climate is prealpine with an average annual precipitation of approximately 900 mm and an average temperature of approximately 7 °C (Ceola and others 2014).

The response variables that we aim to predict and extrapolate are water temperature (T), photosynthetic active radiation (PAR), GPP, and ER. Information about such variables is available simultaneously for 12 stream reaches for the period going from January 2013 to June 2014 (Figure 1). Daily metabolic rates of GPP and ER [gO_2_m^−2^d^−1^] have been locally inferred from high-frequency measurements of dissolved oxygen concentration using the single-station approach (Demars and others 2015). Specifically, we adopted the approach described in Segatto and others (2020) in which GPP is assumed linearly dependent on PAR and ER is constant throughout the day. Further details on the dataset can be found in Ulseth and others (2018); Segatto and others (2020), and in the *Supporting Information (SI) Methods*.

### Random Forest

Random forest (RF) is an ensemble learning algorithm combining many classification or regression trees (CARTs, see Breiman and others 1984) which has been developed to overcome some of the limitations of single CARTs: their poor predictive power and the fact that they do not generalize well from the training data (that is, overfitting, Bramer 2007). One such improvement is bootstrap aggregating, also called bagging (Breiman 1996), which increases the stability and accuracy of CARTs and avoids overfitting. In bagging, multiple trees are trained independently on each other. Trees are created (up to a user-defined number *N*_tree_) by drawing a random subset of training samples with replacement (bootstrap sample). About two-thirds of the sample (referred to as in-bag sample) is typically used to train the single trees. Observations in the original dataset that do not occur in a bootstrap sample are called out-of-bag observations (OOB observations) and are used to estimate the prediction error (typically the root mean square error, RMSE, in case of regression) of the tree (out- of-bag error, OOB error). Random forests (Breiman 2001) are a slightly modified version of bagged trees in which, at each node, only a small number of randomly selected variables (*M*_try_, typically set to one-third of the features for regression trees, Gislason and others (2006)), are made available for the split. In both cases (bootstrap aggregating and RF), the response variable is calculated averaging, in the case of regression, the predictions of all trees. RF turns out to perform very well compared to many other classifiers including popular support vector machines and neural networks and is robust against overfitting (Breiman 2001). To assess the importance of a specific predictor in a RF, the values of that variable are randomly permuted for the OOB observations, and then the modified OOB data are passed down the tree to get new predictions (Breiman 2001). The difference between the RMSEs for the modified and original out-of-bag data is a measure of the importance of the variable. Overfitting can be reduced by selecting only features that improve prediction performances of unseen observations.

### Feature Variables

We defined the stream reach (that is, the channel segment between two consecutive confluences or between the head of a stream and the next confluence), and its underlying subcatchment, as the basic unit of this study. The Ybbs network consists of 292 reaches. An entry for the training of the RF can be established only if all features (or predictors, or learners, that is measurable properties of the phenomenon under study) and response variables (that is, the recorded observations of that phenomenon) are simultaneously available for the same reach and time. A full list of all features used in the RF to predict the response variables is reported and further described in Table S1 in *SI Methods*, while a summary is given in what follows.

Discharge was measured at the catchment closure (Figure 1), and was downscaled to every reach of the network by assuming local discharge proportional to contributing area (Segatto and others 2020). The geomorphic properties of the Ybbs network have been extensively studied by Ceola and others (2014), and we adopted the same exponents to characterize how stream width *w* [m] and the Gauckler Strickler’s roughness coefficient *K*_*s*_ [m^1/3^s^−1^], scale as a power-law function of the contributing area A [km^2^]. We determined the average streambed slope of each reach from the DEM and estimated the time series of mean water depths, z(t) [m], assuming rectangular reach cross sections of constant width and uniform flow conditions (Manning equation, as in Segatto and others (2020)). Few other geomorphological network properties such as distance to the outlet and total upstream length have also been added (*SI Methods*).

We further included spatial information on vegetation cover obtained starting from high-resolution layers of dominant leaf type (DLT), describing the presence or absence of broadleaved or coniferous trees, and tree cover density (TCD) obtained via the Copernicus Land Monitoring Service (Figure 1). Information about surface light exposition has been derived from the DEM by creating a grayscale normalized representation of the catchment exposition, with the sun’s relative position taken into account for shading the image (Figure 1). Starting from the distributed information, we derived both reach vegetation coverage (that is, the average DLT and TCD over the reach pixels) as a proxy of stream canopy shading (termed DLT Local and TCD Local) and watershed vegetation coverage (that is, the average DLT and TCD over the entire subcatchment) as a possible proxy of substrate and nutrients availability (termed DLT WS and TCD WS). The same procedure was followed also for light exposure.

We integrated climatic information using data collected at the weather station in Lunz am See (Figure 1), which included precipitation [mm] and its cumulative duration [min], air temperature [C], barometric pressure [mmHg], irradiation [W m^−2^], duration of sunshine [min] and some derived smoothed signals from temperature (see Figure 1 and Table S1 in *SI Methods*). Moreover, we included distance from the meteo-station to account for possible spatial correlation between variables measured there and in other places of the network. Time-related predictors such as flags tracking the fraction of the day and of the year, month, and season have been added to the feature library. Finally, we implemented the algorithms in Meeus (1991) to predict for each day, the apparent (refraction corrected) sunrise, noon, and sunset times in seconds from midnight and from which we characterized intra-daily expected solar temporal dynamics (see *SI Methods*).

Finally, to possibly account for biophysical properties not explicitly considered, or not available for all reaches, we embodied geometrical features along which such properties could cluster. Specifically, we included Euclidean (that is the subcatchment Cartesian coordinates) and network (that is distance from the outlet) geometrical features.

### Model Training Procedure

We trained the RF for PAR at daily timescale (12 sites × 609 d = 7308 observations/feature; 24 features, (Table S1 in *SI Methods*) and the RF for T at 1 h (7308 × 24 h = 175392 observations/feature; 33 features, Table S1 in *SI Methods*). RFs for GPP and ER have been trained at daily scale as it corresponds to the scale of the single station estimates. Moreover, we investigated the gain in performance that can be achieved including the RF estimates of mean daily T and PAR in the feature portfolio of GPP and ER RFs. After the first set of trials to appreciate the relationship between OBB error and the number of trees *N*_tree_, we set *N*_tree_ = 500 for daily-scale RFs and *N*_tree_ = 200 for the hourly-scale RF, whereas *M*_try_ has been fixed, for all the following scenarios, to the customary value of one-third of the features number (see “Random Forest” section).

We devised two training setups, termed **S** and **T**, designed to probe the performance of the developed RF to extrapolate in space and time, respectively. In training **S**, we assessed the RF spatial prediction power by completely excluding, one at a time, a monitored reach site from the learning stage of each response variable, and evaluating model performance as the RMSE in the predicted response variable in the excluded site. This setup thus implies training 12 RFs (as many as the number of sites) for each response variable. The RFs were originally trained using all available features that were then ranked according to their cumulative OOB variable importance (see “Random Forest” section) over the 12 RFs. The optimal subset of features ensuring nonoverfitting was then selected as follows. The RFs were then retrained multiple times progressively excluding features from the least to the most important. Finally, we selected the subset of features that minimized the RMSE of the prediction of the response variables in the excluded sites (see Table S1 and Figures S1–S6 in *SI Methods*). When extrapolating RF results to the whole river network (292 reaches), we averaged the prediction of the 12 RFs (termed ensemble RF).

To test for the temporal prediction power, we trained a single RF per response variable in which the training dataset included the first year of the time series of all the 12 sites (January 2013–January 2014), while excluding the last six months (named training **T**). Model performance was evaluated as the RMSE in the excluded months of all sites together. Feature selection was then performed as described for training **S** (see Table S1 and Figures S7–S12 in *SI Methods*). Although other configurations are possible, for example simultaneously leaving out a particular site and a particular time span for training, lack of distributed data prevented the investigation of more complex scenarios as the resulting training set would not have left enough information to properly train the model.

Contrarily to the **S** training, we observed that RFs in the **T** setup achieved a low predictive error with few features variable and were not very sensitive to the further addition of features (Figures S7–S12 in *SI Methods*). To have a unique set of features for each response variable, we retained the predictors selected under the more demanding training **S** (see Table S1 in *SI Methods*), and using only the selected features, we repeated the whole training sequence illustrated above (that is, ranking of feature importance, re-training progressively excluding least importance features) to check for possible residual overfit (Figures S13–S22 in *SI Methods*) and evaluate the final predictive error.

### Allochthonous vs Autochthonous Respiration

Combining a carbon removal model with the scaling theory of fractal river network, Bertuzzo and others (2017) predicted that the respiration of allochthonous (that is, coming from the terrestrial ecosystem) material should exhibit a power-law scaling with the drainage area. We combined such prediction with the RF results to provide a first approximation of the separation of the total ER into its allochthonous (ER_al_, that is, terrestrial deliveries to the stream network) and autochthonous (ER_au_, that is, of organic material produced within the network) components. Specifically, we determined the exponent of the power-law scaling via a quantile regression of daily ER values in the different reaches assuming that the lower bound represents the ER_al_ and that any additional respiration should come from ER_au_. We repeated the procedure for four different seasons to capture the possible effect of temperature and delivery rates on ER_al_ (further details in Figure 6).

## Results

### RF Modeling in the Ybbs River Network

Training **T** consistently achieved lower predictive error than training **S** for all response variables (Table 1). Although the latter uses a higher fraction of data (11/12 versus 2/3), this result was expected because training **S** has only 11 sites to learn how to extrapolate spatial features to the whole network. In contrast, training **T** relies on more than 365 time points to learn to predict temporal patterns. We retained the results of training **S** which is deemed more demanding yet more suitable for spatial extrapolation (to which all figures and results refer). A compendium of site-by-site RF predictions for all the implemented training schemes (that is, RF for PAR, T, GPP, ER, and GPP and ER including extrapolated T and PAR under both training **T** and **S** setups) is available in *SI Results* in Figures S23–S30. Partial dependence plots showing the isolated effect of each predictor for each response variable are reported in *SI Results* in Figures S31–S34.

**Table 1:** RF Model Efficiency.

Response Variable	Training **S**^*a*^ NRMSE^*b*^ (%)	Training **T** NRMSE^*b*^ (%)
PAR	6.8	4.8
T	5.5	5.0
GPP	7.8	7.1
ER	9.9	7.7
GPP including T, PAR	7.2	7.1
ER including T, PAR	8.9	7.2

^a^Figures displayed in “Results” section refer to this setup.

^*b*^*Error estimates refer to the RMSEs on the predicted data not used during training, normalized by the range (that is, the difference between the maximum and minimum value) of the measured data. Lower values indicate less residual variance. See SI Results for the single sites prediction errors.*

**Figure 2:**
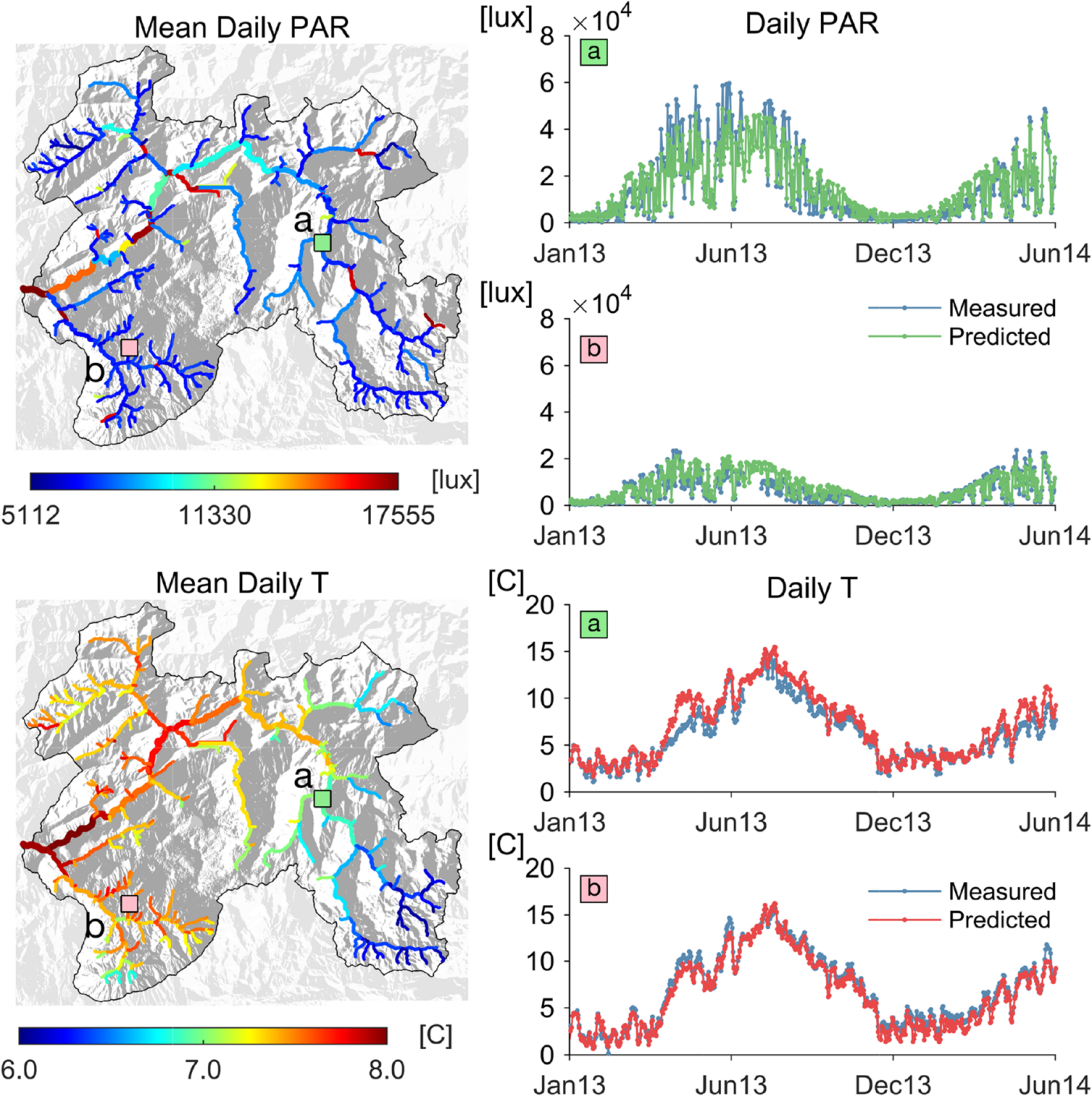
Random forest (RF) predictions of light (PAR) and water temperature (T). Maps in the left column show RF predictions for mean daily PAR (top) and streamwater temperature (bottom) of the 292 stream reaches composing the Ybbs river network. Plots in the right column show the comparison between the time series of measured and predicted mean daily PAR and T for two representative reach sites (a and b, see location on the maps). The comparison for the remaining sites is reported in SI Results in Figures S23 and S25. Results refer to the S training (see “Methods” section). The predicted time series reported here are derived using the RFs trained excluding the site shown (that is, site a and b, respectively). Maps show the results of the ensemble RF, that is, the average of the 12 RFs obtained excluding all sites one at a time (see “Methods” section).

The trained RF correctly recognized upstream reaches with low PAR because of high tree cover density compared to the wider downstream reaches with higher PAR (Figure 2). PAR was heterogeneously distributed throughout the Ybbs River network with daily averages ranging from 5112 to 17555 lux. Modeled average annual streamwater temperatures ranged from 6 to 8°C and clearly increased downstream (Figure 2). The temporal regimes of PAR and streamwater temperature have been correctly learned. For instance, predicted daily PAR and streamwater temperature closely followed measured values in two different subcatchments (Figure 2, see *SI Results* for all other sampled reaches time series and related prediction errors).

**Figure 3:**
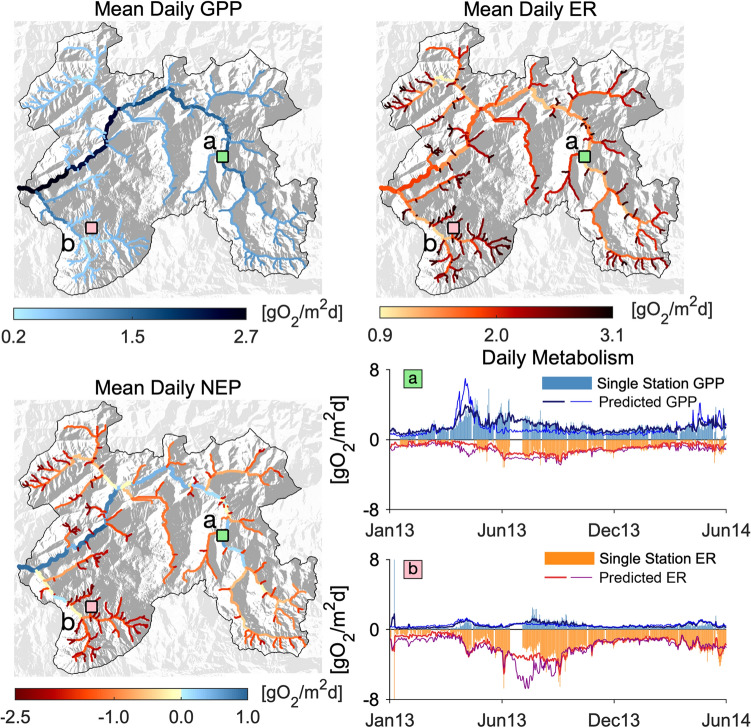
Random Forest (RF) predictions of network-scale metabolic regimes. Maps show RF predictions for mean daily GPP (top-left) and ER (top-right), and NEP (bottom-left) as difference between GPP and ER. Plots (bottom-right) show the comparison between the time series of estimated (via the single-station approach) and predicted (via RF) daily GPP and ER for two representative reach sites (a and b, see location on the maps). The comparison for the remaining sites is reported in SI Results in Figure S27. Results refer to the RF trained in the S setup and that does not include the predicted PAR and T in the feature library (see “Methods” section). Maps show the results of the ensemble RF, that is the average of the 12 RFs obtained excluding all sites one at a time (see “Methods” section). Plots report both the prediction of the ensemble RF (tick line) and of the RF trained excluding the site shown (thin line).

Overall, the RF model reliably predicted the annual regimes of ecosystem GPP and ER and, by difference, also NEP across the Ybbs River network (Figure 3, Table 1). However, we found that including modeled daily PAR and streamwater temperature (Figure 2) did not significantly improve our RF predictions of both GPP and ER regimes. We, therefore, retained the simpler formulation that does not include them in the library of predictive features. Daily metabolism predicted by RF agreed reasonably well with GPP and ER computed from the single-station method as indicated by normalized root-mean square errors (GPP: 5.8 to 18 %; ER: 11 to 18 %) (Figure 3, see *SI Results* for all other sites). Furthermore, we found good agreement between reach-scale mean annual GPP and ER computed from the single-station approach and the RF predictions when the focus site was excluded from training (that is, training **S**) (GPP: *r* = 0*.*7, *p* < 0*.*05; ER: 0.6, *p* < 0*.*05, across the 12 streams within the Ybbs River network, Figure S35 in *SI Results*). The main predictors for GPP were precipitation, irradiation, sunshine duration and period of the year, air temperature and pressure, discharge, streamwater depth, position along the river network, and light exposure. The main predictors for ER were irradiation, sunshine and precipitation duration, period of the year, air temperature and pressure, discharge, slope, length, flow depth, position, and watershed tree cover density and dominant leaf type (see Table S1 in *SI Methods*). RF predicted increasing patterns of mean daily GPP moving from the smallest headwaters (0.2 to almost 1 g O_2_ m^−2^d^−1^), toward the outlet (2.7 g O_2_ m^−2^d^−1^, Figure 3). By contrast, mean daily ER was highest in the headwaters (up to 3.1 g O_2_ m^−2^d^−1^), lowest in the mainstem within the upper catchment (around 0.9 g O_2_ m^−2^d^−1^) and intermediate (around 1.5 g O_2_ m^−2^d^−1^) at the outlet of the Ybbs River network. Calculated mean daily NEP values consistently indicate heterotrophy (-2.5 to -0.5 g O_2_ m^−2^d^−1^) within the headwaters and autotrophy (up to 1 g O_2_ m^−2^d^−1^) along the mainstem, particularly toward the most downstream reaches.

### Spatiotemporal Variation of Ecosystem Metabolism at Network Scale

**Figure 4:**
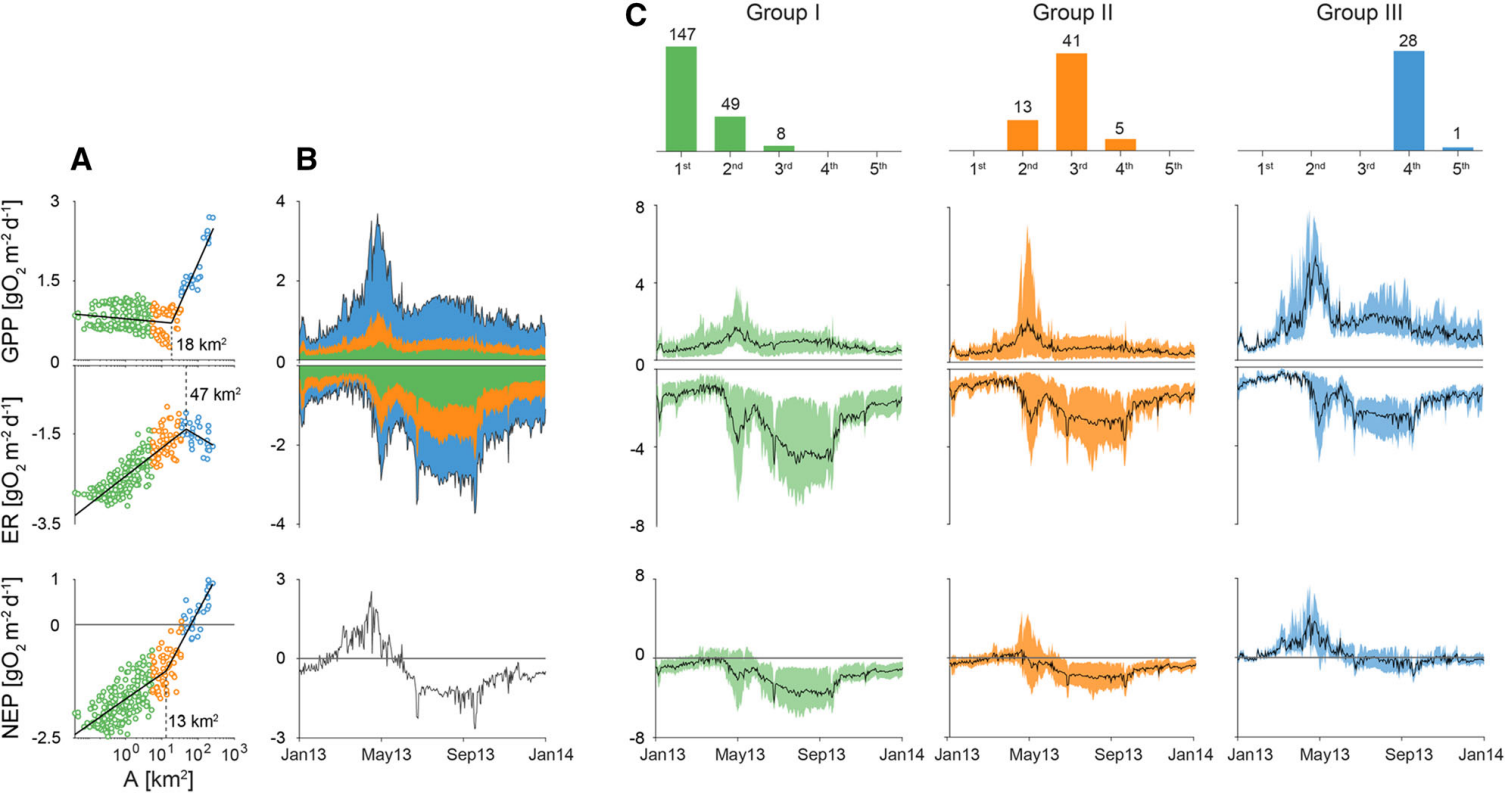
Stream ecosystem metabolic regime at network scale. Stream reaches have been clustered in three major groups according to their Strahler’s stream order and catchment size (panel a and c). Group I (green) includes all streams with catchment size smaller than the largest first-order stream (5.6 km^2^), group II (orange) those smaller than the largest third-order stream (that is, from 5.6 to 36 km^2^) and group III (blue) all the other larger streams. Panel a displays scatter plots of reach-scale mean daily GPP, ER and NEP against drainage area, in logarithmic scale, and their piece-wise regression lines whose equations read as follows (lbp = left side; rbp = right site of the break point): GPP_lbp_ = 0.779 − 0.027 log(A); GPP_rbp_ = −1.264 + 0.676 log(A); ER_lbp_ =  − 2.43 + 0.270 log(A); ER_rbp_ = − 0.59 − 0.207 log(A); NEP_lbp_ = − 651 + 0.240 log(A); NEP_rbp_ =  −2.712 + 0.649 log(A). Breakpoints have been selected to minimize the overall RMSE among all possible combinations (including no breakpoints). Panel b displays the contribution of the different groups to the total, network-scale GPP and ER, expressed per unit of streambed area of the entire river network. Bottom plot of panel b shows network scale NEP per unit of river network streambed area. Top row of panel c shows the frequency distribution of the Strahler’s order of the streams belonging to the three different groups. Bottom plots show the range of variability (colored areas) and the average trend (black lines) of the reach-scale GPP, ER, and NEP of the three different groups.

Integrating the reach-scale metabolic fluxes, we were able to assess ecosystem metabolic regime at the network scale and this across an entire year (Figure 4). This approach detected a conspicuous peak in GPP during spring (April, May), which transiently rendered the entire network metabolism autotrophic with a cumulative NEP of 0.1 Gg O_2_ (equivalent to an areal flux of ∼3 g O_2_ m^−2^d^−1^) during that window (Figure 4B). ER was elevated during summer, which reversed the network-scale metabolism to heterotrophy during the rest of the year. This high level of heterotrophy becomes obvious when comparing the metabolic fluxes cumulated over the entire year and across the 292 streams, with GPP, ER, and NEP averaging 326 (95% range: 173 ÷ 576), −824 (95% range: −1068 ÷ −476), and -498 (95% range: −824 ÷ 107) g O_2_ m^−2^y^−1^, respectively. These areal fluxes translate into network-scale annual fluxes of 0.63, −0.83, and −0.20 Gg O_2_ for GPP, ER and NEP, respectively (Table 2).

**Table 2:** Whole River Network Annual Ecosystem Metabolism Along with the Contribution of the Three Groups Identified.

	Streambed area [km^2^]	GPP [GgO_2_]	ER [GgO_2_]^*b*^	NEP^*a*^ [GgO_2_]
River Network	1.30	0.63	0.83	− 0.20
Group I (n = 204^*c*^)	0.34 (26.2%^*d*^)	0.10 (16.2%)	0.28 (33.7%)	− 0.18
Group II (n = 59)	0.42 (32.1%)	0.13 (20.2%)	0.25 (29.9%)	− 0.12
Group III (n = 29)	0.54 (41.7%)	0.40 (63.6%)	0.30 (36.4%)	0.10

^*a*^NEP, calculated as the difference between GPP and ER.

^*b*^GgO_2_: Giga-grams of O_2_ = 10^9^ gO_2_.

^*c*^N, refers to the number of reaches included in the ith group.

^*d*^*Percentages between parenthesis refer to the relative contribution of the single group to the river network values*.

To assess the relative contributions of small and larger streams to the network metabolism, we aggregated streams into three groups according to their Strahler’s order and the related catchment size. Group I includes all streams with catchment size smaller than the largest first-order stream (5.6 km^2^), group II those smaller than the largest third-order stream (that is, from 5.6 to 36 km^2^), and group III all the other larger streams. Figure 4B shows how the annual, network scale metabolic regime is partitioned among the three groups, while Figure 4C reports the variability of the metabolic patterns among individual reaches belonging to the three different groups. Not unexpectedly, larger streams contributed more than 60% to the network annual GPP despite their smaller contribution by streambed area extent (Table 2). Smallest streams (that is, group I) contributed 26% of the total streambed area, but only 16% of the annual primary production. Interestingly, streams from all three groups contributed more evenly to the network ER (Table 2). As a result of GPP and ER patterns across the network, the smallest streams largely drove the heterotrophy (negative NEP) that we observed at the network scale. A further way to explore stream metabolism across the network is to establish scaling relationships between metabolic fluxes of stream reaches and their position along the river network, here indicated by their catchment size (Figure 4A). Annual mean GPP did not change significantly with catchment size below 18 km^2^, but beyond this threshold GPP significantly increased with the logarithm of catchment size. On the other hand, annual mean ER increased (that is, decreased in absolute value) with catchment size up to 47 km^2^ and decreased beyond this breakpoint. Finally, annual mean NEP increased (that is, became less heterotrophic) with catchment size up to a breakpoint at 13 km^2^, beyond which it increased at an even higher rate, eventually becoming positive toward the outlet.

**Figure 5:**
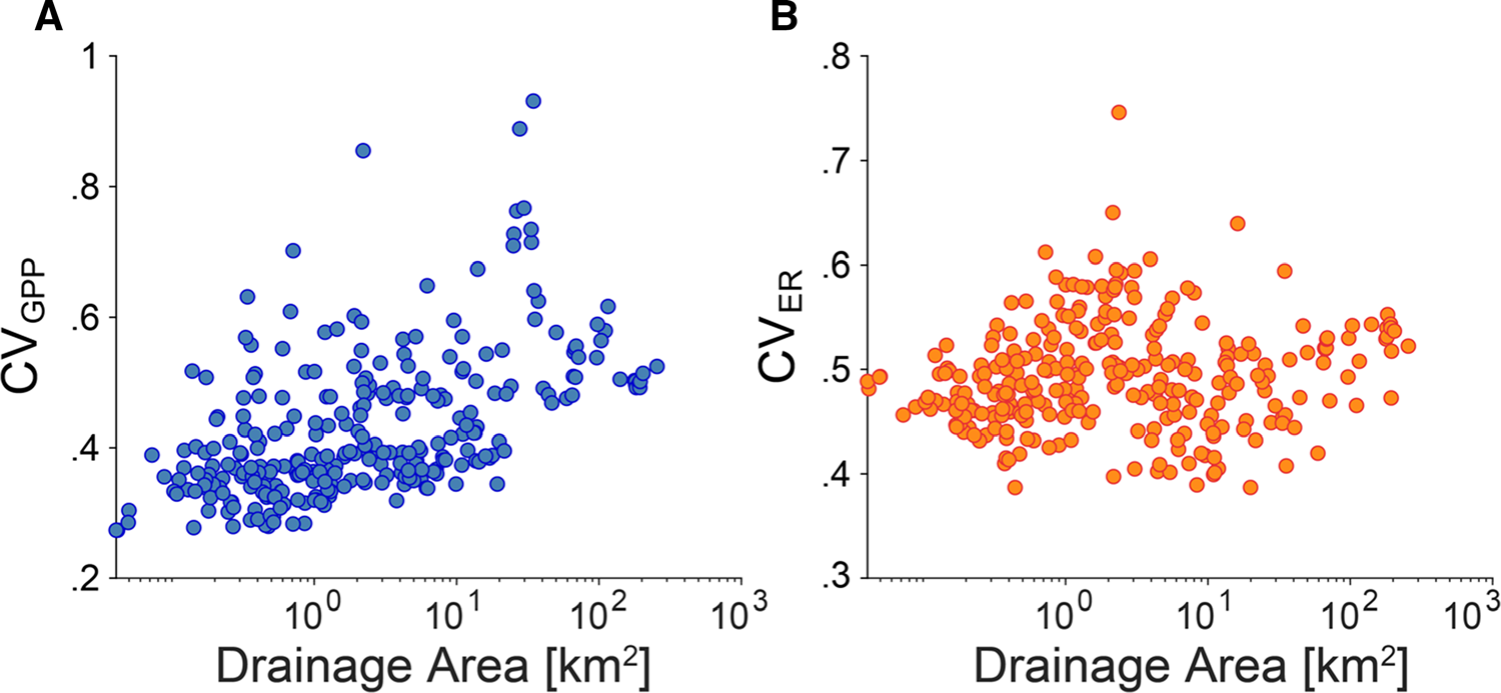
Variability of GPP and ER time series. Coefficient of variation (CV) of the predicted time series of reach-scale GPP (A) and ER (B) as a function of drainage area (log-scale). Each dot represents the CV of one stream reach.

It is relevant to understand the stability of ecosystem processes as a property that potentially emerges from stream networks (for example, Sabo and others 2010; Terui and others 2018). Our predictions from RF modeling allowed us to assess the temporal variability of the metabolic fluxes across the Ybbs River network (Figure 5). Measured as the coefficient of variation (CV) of the daily fluxes over one year, we found that the temporal variability of GPP increased downstream (Pearson’s *r* = 0.33, *p* < 0.01), whereas the temporal variability of ER exhibits a weaker linear downstream trend (*r* = 0.13, *p* < 0.05).

**Figure 6:**
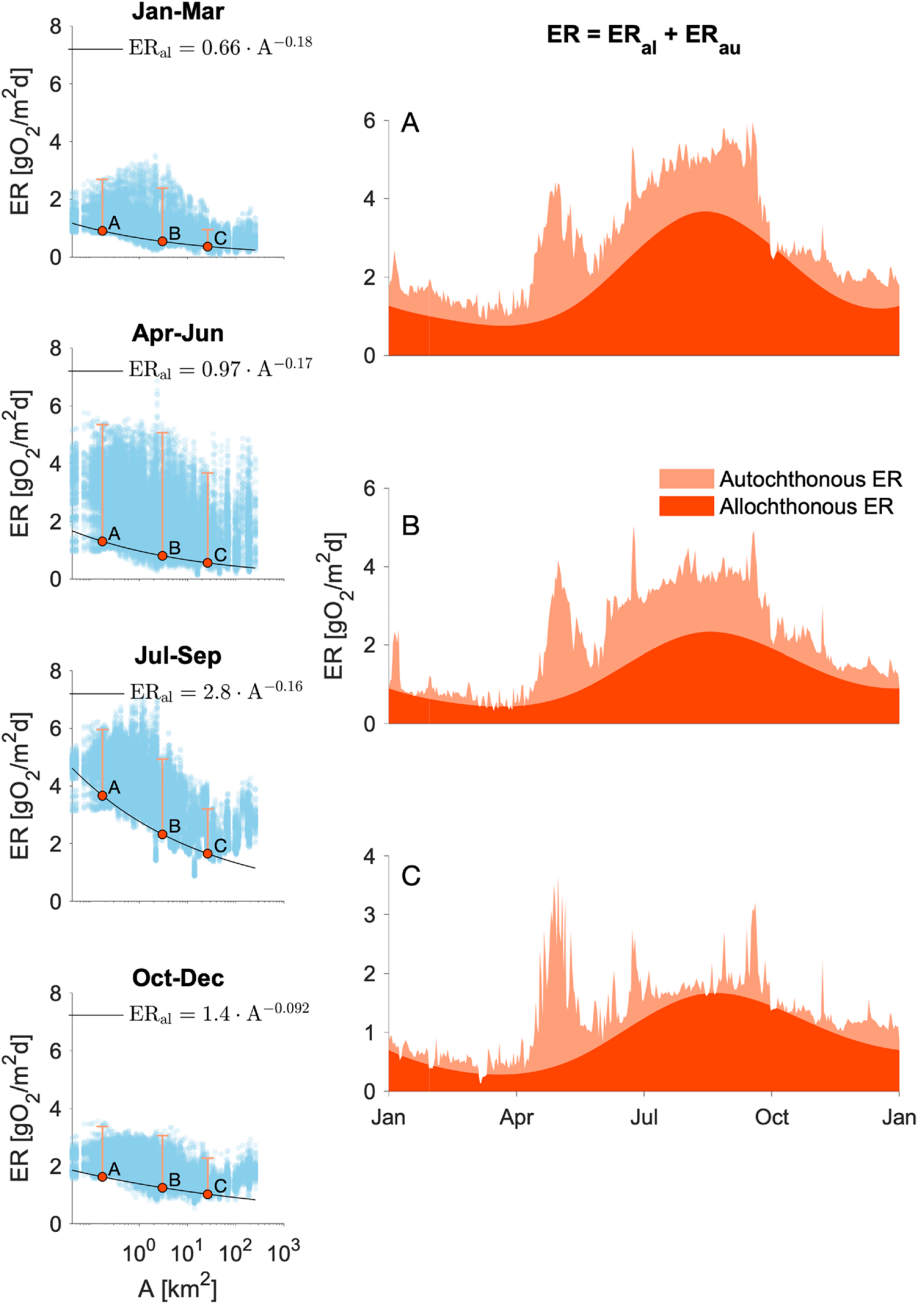
Separation between allochthonous (ER_al_) and autochthonous (ER_au_) ecosystem respiration. Left panels show scatter plots of extrapolated (via RF) daily ER [gO_2_m^2^d^−1^], during the four indicated seasons and in all 292 reaches, as a function of the reach drainage area (A [km^2^]). We determined the exponent of the power law scaling via a 0.05-quantile regression of daily ER values in the different reaches and days assuming that the lower bound represents the ER_al_ and that any additional respiration should come from ER_au_. The ER_al_ time series for each reach has then be derived via a spline interpolation of the four seasonal values. Example for the final separation results is shown in the right panels for one randomly selected reach (A–C) per group (I–III).

**Figure 7:**
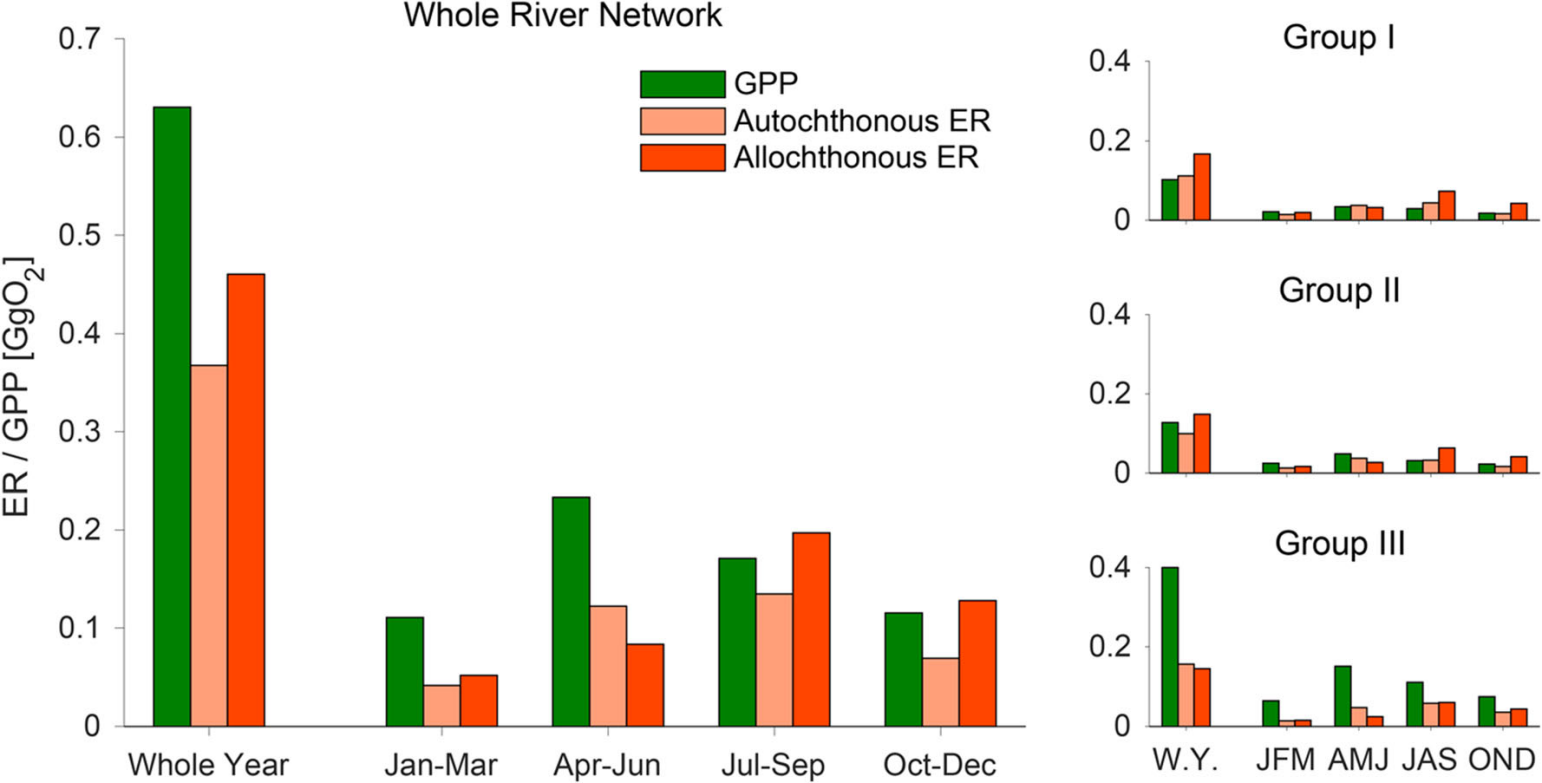
Autochthonous vs allochthonous ecosystem respiration. Comparison between cumulative (mass units, GgO_2_) GPP, autochthonous ER and allochthonous ER, throughout the entire year and at seasonal scale. The main panel displays quantities at the river network scale, while right panels focus on the three groups of stream reaches introduced in Figure 4.

We further combined the RF modeling results and an organic carbon removal model (Bertuzzo and others 2017) to estimate how daily reach-scale ER is partitioned between the respiration of autochthonous (ER_au_, that is, produced as GPP within the same reach or upstream) and allochthonous (ER_al,_ that is, carbon imported from the surrounding terrestrial ecosystem) material (Figures 6, 7). In stream ecology, allochthonous could sometimes refer to the ecosystem outside the single reach being analyzed, including the upstream stream ecosystems. We instead highlight that, as the scale of the present study is the whole network, allochthonous here refers to an origin other than the whole stream ecosystem. We found that at the scale of the entire network and over one year, 37% of the GPP is respired (ER_au_ = 0.37 Gg O_2_) and that allochthonous C was the dominant source to ER (ER_al_ = 0.46 Gg O_2_). Clearly, ER_au_ mirrored the seasonal pattern of GPP, with a peak in spring and a minimum in winter, when the potential for GPP and downstream transport thereof was low. ER_al_ peaked instead in late summer fall. At the network scale ER_au_ was lower than ER_al_ except during spring when a switch in the dominant energy source was observed. When breaking the network-scale fluxes down to groups of stream sites (I, II and III), ER_al_ clearly dominated in almost all groups, whereas ER_au_ increased downstream as did GPP. The excess of GPP that is not respired within the river network is mostly produced in group III.

## Discussion

Over the last decade, the River Continuum Concept has fundamentally shaped our conceptual understanding of how ecosystem metabolism may change along the longitudinal continuum from small streams to larger rivers downstream (Vannote and others 1980; Fisher and others 2004). Despite this, we have not been able to reliably predict stream ecosystem metabolism at the scale that is relevant to carbon cycling. Clearly, this is at the scale of stream networks that drain the landscapes. Although this has been achieved with increasing accuracy for terrestrial ecosystems (Field and others 1998; Anav and others 2015), the heterogeneous and nested structure of stream and river networks has encumbered the prediction of their metabolic fluxes on an annual basis. As a consequence, we have not yet been able to assess properties of metabolic regimes potentially emerging from real-world stream networks.

Our study provides a first proof of concept that data-driven machine learning is a valuable tool to predict the annual regime of ecosystem metabolism at the level of an entire stream network. We opted for RF as a flexible algorithm, whose predictive performance can compete with the best supervised learning techniques (for example, artificial neural networks) (Liu and others 2013; Rodriguez-Galiano and others 2015). Overall, RF predicted reasonably well the annual regimes of GPP and ER. We do acknowledge, however, that our approach failed to reconstruct some temporary and localized events of ecosystem metabolic activity; this failure increased the predictive error of the individual site excluded from training. On the other hand, the annual GPP and ER fluxes agreed well with the observed ones (Figures S35 and S36 in *SI Results*). This highlights the advantage of our modeling approach as it allows the continuous appreciation of annual metabolic regimes that are otherwise often prone to missing data. Furthermore, our annual estimates of GPP and ER are closely bracketed by those reported from other headwater streams covering various biomes (for example, Battin and others 2008; Hoellein and others 2013; Rodríguez-Castillo and others 2019). We are therefore confident that RF modeling is a valuable tool to extrapolate metabolic regimes in every reach of an entire stream network.

The Ybbs streamwater temperature regime at the network scale was predicted by a combination of all available predictors (see *SI Methods* in Table S1), while PAR was best inferred using a combination of meteorological (that is, precipitation, sunshine duration, irradiation, and air temperature and pressure), hydrological (discharge, slope, reach length, and water depth) and geomorphological responses (subcatchment position, local DLT, TCD, and light exposure). Both long- and short-term measurements of ecosystem metabolism at reach scale have revealed PAR and flow-induced disturbance as the major drivers of GPP (for example, Mulholland and others 2001; Beaulieu and others 2013; Blaszczak and others 2019). Using spatial statistics, Rodriguez-Castillo and others (2019) retained channel cross-sectional area, nitrate concentration, and algal biomass as predictors for GPP at the network scale. In our study, out of a library of 35 potential predictors (that is, the “learners”), the RF model retained daily sunshine duration, season, and position within the Ybbs River network, but also discharge, water depth, air temperature, irradiation, light exposure, barometric pressure, and precipitation. In the final version of the model selected, we did not retain the modeled PAR and streamwater temperature T as predictors for GPP (and ER) because the efficiency gain was not high enough (Table 1) to justify the use of a more complicated procedure (a cascade of two RFs) and the introduction of predicted features (PAR and T) possibly prone to errors. It should be noted that all the predictors used by the RF generated to predict PAR and T were also fed to the GPP and ER RFs. Therefore, the algorithm was able to learn the complex nonlinear interactions relating the original predictors to PAR and T, and eventually to GPP and ER, without the need to provide PAR and T separately. Predictors retained for ER included, besides position within the Ybbs River network, descriptors of temperature, soil water content (as approximated by rainfall duration), precipitation and discharge, but also watershed tree cover density and type, for instance. We argue that these predictors translate into the catchment potential to produce and deliver organic matter to the streams and further into the potential of stream ecosystem metabolism. This approach allowed us to extend the library of predictors usually employed in metabolism models (see, for example, Demars and others 2015). Overall, most predictors used are relatively straightforward to extract from various repositories of spatial and meteorological data. Flow-related variables throughout the network can be obtained by combining gauging data (or alternatively hydrological modeling) with scaling relationships of hydraulic geometry (Leopold and Maddock 1953).

We stress that the scope of our study is to investigate emerging patterns of the metabolic regime specifically of the Ybbs river network. For this purpose, we gathered all possible features and let the validation step to properly select those relevant. In general, the interactions between network properties and biological predictors (for example, stream elevation, discharge, and vegetation cover) could be different for different network systems. Moreover, we included few spatial features that improved the performance of the model but are specific to this case study, for example the spatial coordinates and the network geometry. Therefore, the trained models we developed herein are not directly transferable to other river networks. However, this first case study provides a road map for the development of similar applications in other contexts. At this stage, any new application would require enough reach-scale metabolic data to be trained. However, when data for several river networks will be available, and recent technological advances suggest this goal is not that far into the future (Appling and others 2018), we envision that it will be possible to train a model to predict the metabolic regime also in ungauged catchments.

### Network-Scale Ecosystem Metabolism

The GPP regime at the network scale exhibited a bimodal pattern with a conspicuous peak during spring and a reduced, second summer peak. Our findings on a real-world stream network corroborate the emerging property of network-scale GPP as recently reported in theoretical optimal channel networks (Koenig and others 2019). Indeed, the bimodal pattern found for the Ybbs River network resembles that obtained by Koenig and others (2019) under the stochastic assignment, that is, the scenario proposed to be more representative of real rivers by the authors. Dissecting this pattern into the contributions of stream reaches of different sizes to network-scale GPP, we found that downstream reaches were mostly responsible for the spring and summer peak in GPP. This pattern was clearly driven by light availability as modulated by channel geomorphology as predicted by the River Continuum Concept (Vannote and others 1980).

Our findings reveal that the annual metabolism of the Ybbs River network is heterotrophic, with the smallest headwaters contributing most to its heterotrophy. This is in line with earlier predictions on stream ecosystem metabolism (Fisher and Likens 1973; Vannote and others 1980) and studies compiling data from a wide suite of stream ecosystems (Battin and others 2008; Hoellein and others 2013). Perhaps less trivial, our findings provide first insights into the contributions of streams by the areal extent and metabolic fluxes beyond the longitudinal dimension. In this context, the disproportionate contribution of small headwater streams (that is, Group I) to ER and hence to NEP highlights their role for metabolic fluxes at the network scale. Besides the fact that headwater streams are often light-limited owing to riparian vegetation (as tree cover density), per unit area they are more tightly connected to the terrestrial environment than wider reaches further downstream. This increases the terrestrial deliveries of organic matter to these streams. Owing to elevated channel slope, bed roughness, and sediment porosity, the hydrodynamic exchange between the surface water and the streambed is typically higher in headwaters than in downstream reaches, which may enhance the respiratory breakdown of allochthonous organic carbon in these headwater streams (Battin and others 2008).

ER is largely associated with the streambed, notably with its hyporheic zone, which may explain why its annual variability (as CV) does not systematically change across the stream network. Looking more in details at the pattern shown in Figure 5b, the variability of ER is greater in reaches of intermediate size (around few km^2^ of drainage area) and lower for both small headwaters and large streams. From a biophysical point of view, this pattern could be explained by the dominant role of hyporheic respiration in headwaters. Indeed, the hyporheic zone has been proposed as a buffer to ecosystem processes (Boulton and others 1998). In large streams instead, the ER regime could be buffered by the availability of autochthonous biomass and damped thermal and hydrological regimes. However, it should be noted that reaches of intermediate size are also more abundant and therefore this pattern could also be induced by systematic sampling bias.

On the other hand, the downstream increase in the annual variation of GPP (Figure 5a) could be attributed to the susceptibility of GPP to changes in light- and flow-induced disturbance (for example, Uehlinger and others 1996; Uehlinger and Naegeli 1998; Bernhardt and others 2018). In fact, the annual light regime becomes more pronounced downstream as channels widen up. Our findings on the annual variability of GPP at network scale complement those that have related variations in discharge and catchment size to food chain length (Sabo and others 2010). In fact, the annual variation of GPP, and hence the basis of the green food chain, changing through a stream network may shape the phenology of herbivores and their productivity (Bernhardt and others 2018; Rüegg and others 2020). Furthermore, our findings suggest that the respiratory breakdown of organic carbon rather than its photosynthetic buildup shapes the stability of metabolic carbon fluxes at the network scale.

At the stream reach, a negative NEP typically informs on the external deliveries of organic carbon required to satisfy the ER in excess to GPP produced in that reach. It does not inform, however, on the sources of such deliveries, which either derive from the terrestrial environment (that is, allochthonous sources) or from GPP within upstream reaches. This latter would still be considered as an autochthonous source. Combining results from RF modeling with network scaling theory (Bertuzzo and others 2017), we segregated the contributions from allochthonous and autochthonous sources to ER at the network level. This allowed us to assess the relevance of terrestrial subsidies as an energetic linkage between ecosystems (sensu Polis and others 1997). GPP consistently exceeding ER_au_ at the network level indicates that up to 45% of GPP is either exported from the network, potentially fueling metabolism further downstream, or transferred to higher trophic levels. Such a production excess was particularly pronounced during the spring peak of autotrophy in the downstream reaches. Indeed, we estimated that streams draining catchments up to 36 km^2^ in size (group I and II) respire on an annual basis all the organic carbon produced therein (Figure 7).

Strikingly, our findings on ER_al_ suggest that stream networks are more heterotrophic than hitherto assumed by simply looking at NEP. In fact, while the annual network-scale NEP is -0.2 g O_2_ m^−2^d^−1^, the corresponding ER_al_ is -0.46 g O_2_ m^−2^d^−1^ (Figure 7), the difference being due to the GPP not respired within the network. This difference becomes even more pronounced for the small streams in fall and winter (Figure 7). We interpret this finding as evidence for strong ecosystem-level allochthony in streams, in analogy to the lake allochthony (Carpenter and others 2005), and otherwise explored at the level of food webs in streams (for example, Collins and others 2016). Furthermore, these findings evoke a high retention efficiency of individual reaches. In fact, GPP fuels the heterotrophic metabolism because of the typically high bioavailability of algal exudates and the close spatial proximity of algae and heterotrophs within benthic biofilms (for example, Haack and McFeters 1982; Kaplan and Bott 1989). Our ecosystem-level observations that a large proportion of GPP is respired are indeed supported by experimental work on photoautotrophic biofilms from the Ybbs River network (Oberer Seebach) showing that benthic community respiration is sustained between 60 and 90% by organic carbon exuded from algae (Wagner and others 2017).

In this study, we have shown how network-scale applications to stream ecosystem metabolism represent the cornerstone for unveiling metabolic emerging properties, scaling relationships, and to properly assess the relative contributions of small and large streams to GPP, ER, and NEP fluxes at scales relevant for regional carbon budgets. We thus believe that this approach opens new possibilities in modeling and estimating stream ecosystem metabolism. For example, reach-scale models leveraging on the knowledge of the extrapolated forcings (for example, light and temperature) can be developed to provide a more process-based interpretation of network-scale metabolism. Moreover, climate change implications, for instance of more extreme thermal and hydrological regimes, could be more reliably tested considering the entire river continuum. Finally, we anticipate that other emerging properties for metrics like ecosystem efficiency, carbon turnover times, and carbon spiraling (Newbold and others 1982; Webster and Meyer 1997) could possibly be unveiled and tested zooming out the analysis at the scale of an entire stream network.

## Supplementary Information

Below is the link to the electronic supplementary material.

Supplementary file1 (PDF 11,550 kb)

## Data Availability

All input data and model results are available at the following public repository: 10.5281/zenodo.3972937.

## References

[CR1] Anav A, Friedlingstein P, Beer C, Ciais P, Harper A, Jones C, Murray-Tortarolo G, Papale D, Parazoo NC, Peylin P (2015). Spatiotemporal patterns of terrestrial gross primary production: A review. Reviews of Geophysics.

[CR2] Appling AP, Read JS, Winslow LA, Arroita M, Bernhardt ES, Griffiths NA, Hall RO, Harvey JW, Heffernan JB, Stanley EH (2018). The metabolic regimes of 356 rivers in the united states. Scientific data.

[CR3] Battin TJ, Besemer K, Bengtsson MM, Romani AM, Packmann AI (2016). The ecology and biogeochemistry of stream biofilms. Nature Reviews Microbiology.

[CR4] Battin TJ, Kaplan LA, Findlay S, Hopkinson CS, Marti E, Packman AI, Newbold JD, Sabater F (2008). Biophysical controls on organic carbon fluxes in fluvial networks. Nature Geoscience.

[CR5] Battin TJ, Luyssaert S, Kaplan LA, Aufdenkampe AK, Richter A, Tranvik LJ (2009). The boundless carbon cycle. Nature Geoscience.

[CR6] Beaulieu JJ, Arango CP, Balz DA, Shuster WD (2013). Continuous monitoring reveals multiple controls on ecosystem metabolism in a suburban stream. Freshwater Biology.

[CR7] Beer C, Reichstein M, Tomelleri E, Ciais P, Jung M, Carvalhais N, Rödenbeck C, Arain MA, Bal- docchi D, Bonan GB, and others, (2010). Terrestrial gross carbon dioxide uptake: global distribution and covariation with climate. Science.

[CR8] Bernhardt ES, Heffernan JB, Grimm NB, Stanley EH, Harvey J, Arroita M, Appling AP, Cohen M, McDowell WH, Hall R (2018). The metabolic regimes of flowing waters. Limnology and Oceanography.

[CR9] Bertuzzo E, Helton AM, Hall RO, Battin TJ (2017). Scaling of dissolved organic carbon removal in river networks. Advances in water resources.

[CR10] Besemer K, Singer G, Quince C, Bertuzzo E, Sloan W, Battin TJ (2013). Headwaters are critical reservoirs of microbial diversity for fluvial networks. Proceedings of the Royal Society B: Biological Sciences.

[CR11] Blaszczak JR, Delesantro JM, Urban DL, Doyle MW, Bernhardt ES (2019). Scoured or suffocated: Urban stream ecosystems oscillate between hydrologic and dissolved oxygen extremes. Limnology and Oceanography.

[CR12] Botter G, Basso S, Rodriguez-Iturbe I, Rinaldo A (2013). Resilience of river flow regimes. Proceedings of the National Academy of Sciences.

[CR13] Boulton AJ, Findlay S, Marmonier P, Stanley EH, Valett HM (1998). The functional significance of the hyporheic zone in streams and rivers. Annual Review of Ecology and Systematics.

[CR14] Bramer M 2007. Principles of data mining volume 180. Springer.

[CR80] Breiman L (1996). Bagging predictors. Machine Learning.

[CR15] Breiman L (2001). Random forests. Machine learning.

[CR16] Breiman L, Friedman J, Olshen R, Stone C 1984. Classification and regression trees. wadsworth int. Group 37(15): 237–51.

[CR17] Carpenter SR, Cole JJ, Pace ML, Van de Bogert M, Bade DL, Bastviken D, Gille CM, Hodgson JR, Kitchell JF, Kritzberg ES (2005). Ecosystem subsidies: terrestrial support of aquatic food webs from 13c addition to contrasting lakes. Ecology.

[CR18] Ceola S, Bertuzzo E, Singer G, Battin TJ, Montanari A, Rinaldo A (2014). Hydrologic controls on basin-scale distribution of benthic invertebrates. Water Resources Research.

[CR19] Chapin FS, Woodwell GM, Randerson JT, Rastetter EB, Lovett GM, Baldocchi DD, Clark DA, Harmon ME, Schimel DS, Valentini R (2006). Reconciling carbon-cycle concepts, ter- minology, and methods. Ecosystems.

[CR20] Collins SM, Kohler TJ, Thomas SA, Fetzer WW, Flecker AS (2016). The importance of terrestrial subsidies in stream food webs varies along a stream size gradient. Oikos.

[CR21] Demars BO (2019). Hydrological pulses and burning of dissolved organic carbon by stream respiration. Limnology and Oceanography.

[CR22] Demars BO, Thompson J, Manson JR (2015). Stream metabolism and the open diel oxygen method: Principles, practice, and perspectives. Limnology and Oceanography: Methods.

[CR23] Drake TW, Raymond PA, Spencer RG (2018). Terrestrial carbon inputs to inland waters: A current synthesis of estimates and uncertainty. Limnology and Oceanography Letters.

[CR24] Duvert C, Butman DE, Marx A, Ribolzi O, Hutley LB (2018). Co 2 evasion along streams driven by groundwater inputs and geomorphic controls. Nature geoscience.

[CR25] Falkowski PG, Barber RT, Smetacek V (1998). Biogeochemical controls and feedbacks on ocean primary production. Science.

[CR26] Field CB, Behrenfeld MJ, Randerson JT, Falkowski P 1998. Primary production of the biosphere: integrating terrestrial and oceanic components. science 281(5374): 237–40. 10.1126/science.281.5374.237 9657713

[CR27] Fisher SG, Likens GE (1973). Energy flow in bear brook, new hampshire: an integrative approach to stream ecosystem metabolism. Ecological monographs.

[CR28] Fisher SG, Sponseller RA, Heffernan JB (2004). Horizons in stream biogeochemistry: flowpaths to progress. Ecology.

[CR29] Gislason PO, Benediktsson JA, Sveinsson JR (2006). Random forests for land cover classification. Pattern Recognition Letters.

[CR30] Goulden ML, McMillan A, Winston G, Rocha A, Manies K, Harden JW, Bond-Lamberty B (2011). Patterns of npp, gpp, respiration, and nep during boreal forest succession. Global Change Biology.

[CR31] Haack TK, McFeters GA (1982). Nutritional relationships among microorganisms in an epilithic biofilm community. Microbial ecology.

[CR32] Hall RO, Tank JL, Baker MA, Rosi-Marshall EJ, Hotchkiss ER (2016). Metabolism, gas exchange, and carbon spiraling in rivers. Ecosystems.

[CR33] Hall RO, Beaulieu JJ (2013). Estimating autotrophic respiration in streams using daily metabolism data. Freshwater Science.

[CR34] Hall RO, Yackulic CB, Kennedy TA, Yard MD, Rosi-Marshall EJ, Voichick N, Behn KE (2015). Turbidity, light, temperature, and hydropeaking control primary productivity in the c olorado river, g rand c anyon. Limnology and Oceanography.

[CR35] Heimann M, Reichstein M (2008). Terrestrial ecosystem carbon dynamics and climate feedbacks. Nature.

[CR36] Helton AM, Hall RO, Bertuzzo E (2018). How network structure can affect nitrogen removal by streams. Freshwater Biology.

[CR37] Hoellein TJ, Bruesewitz DA, Richardson DC (2013). Revisiting odum (1956): A synthesis of aquatic ecosystem metabolism. Limnology and Oceanography.

[CR38] Horgby Å, Gómez-Gener L, Escoffier N, Battin TJ (2019). Dynamics and potential drivers of co2 concentration and evasion across temporal scales in high-alpine streams. Environmental Research Letters.

[CR39] Horgby Å, Segatto PL, Bertuzzo E, Lauerwald R, Lehner B, Ulseth AJ, Vennemann TW, Battin TJ (2019). Unexpected large evasion fluxes of carbon dioxide from turbulent streams draining the world’s mountains. Nature communications.

[CR40] Hotchkiss E, Hall R, Sponseller R, Butman D, Klaminder J, Laudon H, Rosvall M, Karlsson J (2015). Sources of and processes controlling co 2 emissions change with the size of streams and rivers. Nature Geoscience.

[CR41] Kaplan LA, Bott TL (1989). Diel fluctuations in bacterial activity on streambed substrata dur- ing vernal algal blooms: effects of temperature, water chemistry, and habitat. Limnology and Oceanography.

[CR42] Koenig LE, Helton AM, Savoy P, Bertuzzo E, Heffernan JB, Hall RO, Bernhardt ES (2019). Emergent productivity regimes of river networks. Limnology and Oceanography Letters.

[CR43] Leopold LB, Maddock T 1953. The hydraulic geometry of stream channels and some physiographic implications volume 252. US Government Printing Office.

[CR44] Liu M, Wang M, Wang J, Li D (2013). Comparison of random forest, support vector machine and back propagation neural network for electronic tongue data classification: Application to the recognition of orange beverage and chinese vinegar. Sensors and Actuators B: Chemical.

[CR45] Meeus JH (1991). Astronomical algorithms.

[CR46] Mulholland P, Fellows C, Tank J, Grimm N, Webster J, Hamilton S, Martí E, Ashkenas L, Bowden W, Dodds W (2001). Inter-biome comparison of factors controlling stream metabolism. Freshwater biology.

[CR47] Newbold J, Mulholland P, Elwood J, O’neill R, (1982). Organic carbon spiralling in stream ecosystems. Oikos.

[CR48] Olden JD, Lawler JJ, Poff NL (2008). Machine learning methods without tears: a primer for ecologists. The Quarterly review of biology.

[CR49] Palmer M, Ruhi A (2019). Linkages between flow regime, biota, and ecosystem processes: Implications for river restoration. Science.

[CR50] Polis GA, Anderson WB, Holt RD (1997). Toward an integration of landscape and food web ecology: the dynamics of spatially subsidized food webs. Annual review of ecology and systematics.

[CR51] Raymond PA, Hartmann J, Lauerwald R, Sobek S, McDonald C, Hoover M, Butman D, Striegl R, Mayorga E, Humborg C (2013). Global carbon dioxide emissions from inland waters. Nature.

[CR52] Raymond PA, Saiers JE, Sobczak WV (2016). Hydrological and biogeochemical controls on watershed dissolved organic matter transport: Pulse-shunt concept. Ecology.

[CR53] Regnier P, Friedlingstein P, Ciais P, Mackenzie FT, Gruber N, Janssens IA, Laruelle GG, Lauerwald R, Luyssaert S, Andersson AJ (2013). Anthropogenic perturbation of the carbon fluxes from land to ocean. Nature geoscience.

[CR54] Reichstein M, Camps-Valls G, Stevens B, Jung M, Denzler J, Carvalhais N (2019). Deep learning and process understanding for data-driven earth system science. Nature.

[CR55] Roberts BJ, Mulholland PJ, Hill WR (2007). Multiple scales of temporal variability in ecosystem metabolism rates: results from 2 years of continuous monitoring in a forested headwater stream. Ecosystems.

[CR56] Rocher-Ros G, Sponseller RA, Lidberg W, Mörth CM, Giesler R (2019). Landscape process domains drive patterns of co2 evasion from river networks. Limnology and Oceanography Letters.

[CR57] Rodríguez-Castillo T, Estévez E, González-Ferreras AM, Barquín J (2019). Estimating ecosystem metabolism to entire river networks. Ecosystems.

[CR58] Rodriguez-Galiano V, Sanchez-Castillo M, Chica-Olmo M, Chica-Rivas M (2015). Machine learning predictive models for mineral prospectivity: An evaluation of neural networks, random forest, regression trees and support vector machines. Ore Geology Reviews.

[CR59] Rüegg J, Chaloner DT, Ballantyne F, Levi PS, Song C, Tank JL, Tiegs SD, Lamberti GA (2020). Understanding the relative roles of salmon spawner enrichment and disturbance: A high-frequency, multi-habitat field and modeling approach. Frontiers in Ecology and Evolution.

[CR60] Sabo JL, Finlay JC, Kennedy T, Post DM 2010. The role of discharge variation in scaling of drainage area and food chain length in rivers. science 330(6006): 965–67. 10.1126/science.1196005 20947729

[CR61] Segatto PL, Battin TJ, Bertuzzo E (2020). Modeling the coupled dynamics of stream metabolism and microbial biomass. Limnology and Oceanography.

[CR62] Tank SE, Fellman JB, Hood E, Kritzberg ES (2018). Beyond respiration: Controls on lateral carbon fluxes across the terrestrial-aquatic interface. Limnology and Oceanography Letters.

[CR63] Terui A, Ishiyama N, Urabe H, Ono S, Finlay JC, Nakamura F (2018). Metapopulation stability in branching river networks. Proceedings of the National Academy of Sciences.

[CR64] Uehlinger U, Bührer H, Reichert P (1996). Periphyton dynamics in a floodprone prealpine river: evaluation of significant processes by modelling. Freshwater Biology.

[CR65] Uehlinger U, Naegeli MW (1998). Ecosystem metabolism, disturbance, and stability in a prealpine gravel bed river. Journal of the North American Benthological Society.

[CR66] Ulseth AJ, Bertuzzo E, Singer GA, Schelker J, Battin TJ (2018). Climate-induced changes in spring snowmelt impact ecosystem metabolism and carbon fluxes in an alpine stream network. Ecosys- tems.

[CR67] Vannote RL, Minshall GW, Cummins KW, Sedell JR, Cushing CE (1980). The river continuum concept. Canadian journal of fisheries and aquatic sciences.

[CR68] Wagner K, Bengtsson MM, Findlay RH, Battin TJ, Ulseth AJ (2017). High light intensity mediates a shift from allochthonous to autochthonous carbon use in phototrophic stream biofilms. Journal of Geophysical Research: Biogeosciences.

[CR69] Webster J, Meyer JL (1997). Organic matter budgets for streams: a synthesis. Journal of the North American Benthological Society.

[CR70] Widder S, Besemer K, Singer GA, Ceola S, Bertuzzo E, Quince C, Sloan WT, Rinaldo A, Battin TJ (2014). Fluvial network organization imprints on microbial co-occurrence networks. Proceedings of the National Academy of Sciences.

[CR71] Xiao J, Sun G, Chen J, Chen H, Chen S, Dong G, Gao S, Guo H, Guo J, Han S (2013). Carbon fluxes, evapotranspiration, and water use efficiency of terrestrial ecosystems in china. Agricultural and Forest Meteorology.

