# Supplementary material for: The Metabolic Regimes at the Scale of an Entire Stream Network Unveiled Through Sensor Data and Machine Learning
Source: Ecosystems. 2021 Apr 2;24(7):1792–809. doi: 10.1007/s10021-021-00618-8 (PMC8593893; doi:10.1007/s10021-021-00618-8)
Supplement: Supplementary file 1 — Supplementary file1 (PDF 11,550 kb) [file 10021_2021_618_MOESM1_ESM.pdf]

The metabolic regimes at the scale of an entire stream network unveiled through sensor data and machine learning
Supplementary Information

Pier Luigi Segatto[*]

Stream Biofilm and Ecosystem Research Laboratory
Ecole Polytechinque Fédérale de Lausanne, Lausanne, CH-1015, Switzerland
pier.segatto@epfl.ch

Tom J. Battin[†]

Stream Biofilm and Ecosystem Research Laboratory
Ecole Polytechinque Fédérale de Lausanne, Lausanne, CH-1015, Switzerland
tom.battin@epfl.ch

Enrico Bertuzzo[‡]

Department of Environmental Sciences, Informatics and Statistics
University of Venice Ca' Foscari, 30170 Venice, Italy
enrico.bertuzzo@unive.it

December 14, 2020

We provide in what follows a compendium of the predictors employed in the RF models presented in the main text (see Table S1). Further, we report plots of the error in the predicted sample (excluded from training) as function of the number of predictors used, ranked according to their OOB importance (see Methods section in main text). In the first sub-set of figures (Figure S1-S6) we show the variable selection procedure for RF tested according to training **S** set up. Shaded areas show the variables that contributes to the error minimization and depict the features that have been used to derive results shown in the main text. Then, plots for training **T** are shown (Figure S7-S12). We then chose as final library of features those selected under training **S**, i.e. those minimizing the total error in Figures S1-S6. We thus re-trained RFs under both **S** and **T** set ups using such feature portfolio (see Methods section in main text). A check for possible residual overfit in the final RFs has been carried out in Figure S13-S22 where we repeated the variable selection procedure.

This document then provides plots (Figure S23-S30) of the final RF model outputs of PAR, T, GPP and ER and for each site. We stress that these plots show the goodness of fit of the algorithm in predicting data that were not used in training. Furthermore, partial dependence plots of each feature making the library of each of the final RFs shown in the main text are reported in Figures S31-S34.

Finally, we report plots with a one to one comparison between the RF and single station annual estimates of both metabolic areal fluxes (Figure S35) and total masses (Figure S36) for all twelve sites.

SI Methods - Feature Variables

Selected time-steps:

[*]Lead Author
[†]Corresponding author
[‡]Corresponding author

Table S1: List of learners used for training[a] the specific RFs algorithms. Symbol "✓" has been used when the corresponding feature has been included in the library of the specific RF while symbol "×" when the feature has been excluded.

Feature	Units	PAR	T	GPP	ER
Precipitation	[mm]	✓	✓	✓	✓
Precipitation duration	[min]	✓	✓	✓	✓[c]
Global irradiation	[W/m^2]	✓	✓	✓	✓
Sunshine duration	[min]	✓	✓	✓	✓
Air temperature	[C]	✓	✓	✓	✓
Averaged 2h antecedent air T	[C]	×	✓	×	×
Exp smoothed air T over last 4 h	[C]	×	✓	×	×
Air pressure	[mmHg]	✓	✓	✓	✓
Coord. X	[m]	✓	✓	✓	✓
Coord. Y	[m]	✓	✓	✓	✓
Coord. Z	[m]	✓	✓	✓	✓
Distance from weather station	[m]	✓	✓	✓	✓
Drainage area	[m^2]	✓	✓	✓[b]	✓
Discharge	[m^3/d]	✓	✓	✓	✓
Reach width	[m]	✓	✓	✓[b]	✓
Reach slope	[-]	✓	✓	✓	✓
Reach length	[m]	✓	✓	✓	✓
Stage	[m]	✓	✓	✓	✓
Dist. to outlet	[m]	✓	✓	✓	✓
Tot dist. upstream	[m]	✓	✓	✓	✓
Light exposure WS	[%]	×	✓	✓	✓
Light exposure Local	[%]	✓	✓	✓	✓
DLT WS	[-]	×	✓	✓	✓[c]
DLT Local	[-]	✓	✓	✓	✓
TCD WS	[%]	×	✓	✓	✓[c]
TCD Local	[%]	✓	✓	✓	✓
Fraction of the day	[-]	×	✓	×	×
Lights ON	[-]	✓	✓	×	×
Almost lights ON	[-]	×	✓	×	×
Rising falling sun	[-]	×	✓	×	×
Day of the year	[-]	✓	✓	✓	✓
Month of the year	[-]	✓	✓	✓	✓
Season	[-]	✓	✓	✓	✓
Daily PAR	[lux]	×	×	✓[b]	✓[b]
Daily water T	[C]	×	×	✓[b]	✓[b]

[a] Highlighted cells refer to features that have been retained after model optimization (feature selection, see Figures S1-S22), without including PAR and T extrapolations for GPP and ER and following the 12-forests set-up (training **S**).

[b] Extra Feature retained when adding extrapolated Daily PAR and Daily T.

[c] Extra Feature discarded when adding extrapolated Daily PAR and Daily T.

1. dtD = 1 day (used for PAR, GPP, ER)
2. dtm = 15 min (used for T)

Features description:

1. Precipitation : Cumulative precipitation [mm] over the last dtD or dtm.
2. Precipitation duration : Cumulative precipitation duration [min] over the last dtD or dtm.
3. Global irradiation : average incident irradiation [W/m^2].
4. Sunshine duration : cumulative duration of sunshine [sec] over the last dtD or dtm.
5. Air temperature : average air temperature [C].
6. Averaged 2h antecedent air T : Average air T over the last 2 h. It is used to gain knowledge about the immediate past, i.e. increasing or decreasing expected T trend.
7. Exp smoothed air T over last 4 h: Exponentially smoothed air T using a moving window of 4 h. It is used to gain knowledge about the immediate past, i.e. increasing or decreasing expected T trend.
8. Air pressure : Average barometric pressure [mmHg].
9. Coord. X : x coordinate [m] of the measurement site (when training RF) or of the baricenter of the sub-cathcment (when predicting new data).
10. Coord. Y : y coordinate [m] of the measurement site (when training RF) or of the baricenter of the sub-cathcment (when predicting new data).
11. Coord. Z : z coordinate [m] of the measurement site (when training RF) or of the baricenter of the sub-cathcment (when predicting new data).
12. Distance from weather station : 3D Euclidean distance [m] of the triplet (X,Y,Z) from the weather station of Lunz am See.
13. Drainage Area : Drainage area (A) of the sub-catchment [m^2].
14. Discharge : at the outlet of the sub-catchment (Q(t)), in [m^3/d].
15. Reach width: reach width (w) predicted according to the following scaling relationship: $w = w_{max}(A/A_{max})^{0.44}$, where $w_{max} = 25\ m$ is the maximum stream width measured at the outlet of the catchment and A_{max} is the catchment drainage area (see main text).
16. Reach slope : average slope (i) of the selected reach [-].
17. Reach length : length of the selected reach [m].
18. Stage : water depth (z(t)) [m] derived according to the Manning's equation: $Q(t) = w\ z(t) K_s\ r(t)^{2/3}\sqrt{i}$, where K_s is the Glauckler Strickler's roughness coefficient, calculated according to the scaling relationship $K_s = K_{s,max}(A/A_{max})^{0.35}$ ($K_{s,max} = 20\ m^{1/3}s^{-1}$, estimated value at the outlet); $r(t)$ is the hydraulic radius, i.e., the ratio of the cross-sectional area to the wet perimeter, which for rectangular cross sections reads $r = w \cdot z/(w + 2z)$.
19. Dist. to outlet : hydrological distance from the closure of the sub-catchment to the outlet of the catchment [m].
20. Tot dist. upstream : sum of the length of all the pixels upstream of the sub-catchment closure [m].

21. Light exposure WS : Average light exposure [%] of all the pixels belonging to the considered subcatchment. This variable varies between 0 (subcatchment not exposed to direct light) and 100 (full exposition to direct light). Light exposure has been calculated by creating a grayscale normalized representation of the catchment, with the sun's relative position taken into account for shading the image (`https://www.mathworks.com/matlabcentral/fileexchange/14863-hillshade`). The assumed position is the average sun position at the YRN which is at 90°-Latitude = 42.2° from the horizon (i.e., sun altitude) and 90°, i.e., South (sun azimuth)).

22. Light exposure Local : corresponds to the light exposure [%] of the pixel of the measurement site (when training the RF) or to the average light exposure of all channelized pixels belonging to the considered sub-catchment (when predicting new data). It varies between 0 (no exposition to direct light) and 100 (full exposition to direct light).

23. DLT WS : represents the average dominant leaf type (DLT) [-] over the pixels composing the considered sub-catchment. DLT assumes values between 0 (absence of vegetation), 1 (broadleaved trees) and 2 (coniferous trees). Data have been downloaded from the Copernicus Land Monitoring Service at 30 m resolution. We obtained a 10 m resolution layer using a rotationally symmetric Gaussian lowpass filter of size 50 m and standard deviation 1.

24. DLT Local : corresponds to the DLT [-] of the pixel corresponding to the measurement site (when training the RF) or to the average DLT of all channelized pixels of the considered sub-catchment (when predicting new data). DLT assumes values between 0 (absence of vegetation), 1 (broadleaved trees) and 2 (coniferous trees).

25. TCD WS : represents the average tree cover density (TCD) [%] over the pixels composing the considered sub-catchment. TCD assumes values between 0 (absence of tree cover) and 100 (pixel completely covered). Data have been downloaded from the Copernicus Land Monitoring Service at 30 m resolution. We obtained a 10 m resolution layer using a rotationally symmetric Gaussian lowpass filter of size 50 m and standard deviation 1.

26. TCD Local : corresponds to the TCD [%] of the pixel of the measurement site (when training the RF) or to the average TCD of all channelized pixels of the considered sub-catchment (when predicting new data). TCD assumes values between 0 (absence of tree cover) and 100 (pixel completely covered).

27. Fraction of the day : fraction of the day [-] at which the considered observation has been taken. It varies between 0 (00:00:00) and 1 (23:59:59).

28. Lights ON : We derived sunrise, noon and sunset times in seconds from midnight using the algorithms cited in the main text. Thus, each observation is flagged with 0 [-] if it has been gathered before sunrise or after sunset, 1 otherwise.

29. Almost lights ON : similar to the Lights ON feature. The only difference is that we allowed for a slightly wider window of sunshine (sunrise anticipated of 1.5 h) in order to embed light diffusive effects.

30. Rising falling sun : flag tracking the expected movement of the sun [-] based on calculated sunrise, noon and sunset times (see above). It assumes 0 when time is before sunrise or after sunset, 1 when time is after sunrise but before noon and -1 otherwise (i.e., before sunrise and after noon-time).

31. Day of the year : flag tracking the day of the year [-] (1-365).

32. Month of the year : flag tracking the month of the year [-] (1-12).

33. Season : flag tracking the season of the year [-] (1-4). Spring = 1, Summer = 2, Autumn = 3, Winter = 4.

34. Daily PAR : Predicted PAR [lux] at the considered sub-catchment.

35. Daily T : Predicted T [C] at the considered sub-catchment.

SI Methods - Training Procedure

1 - training S: RF training and feature selection

Figures S1 - S6.

2 - training T: RF training and feature selection

Figures S7 - S12.

3 - Re-training using best predictors of training S and overfit check for both setups

Figures S13 - S22.

SI Results

4 - Final RF Model Output

Figures S23 - S30.

5 - Partial dependence plots.

Figures S31 - S34.

6 - Miscellaneous: single station comparison

Figures S35 - S36.

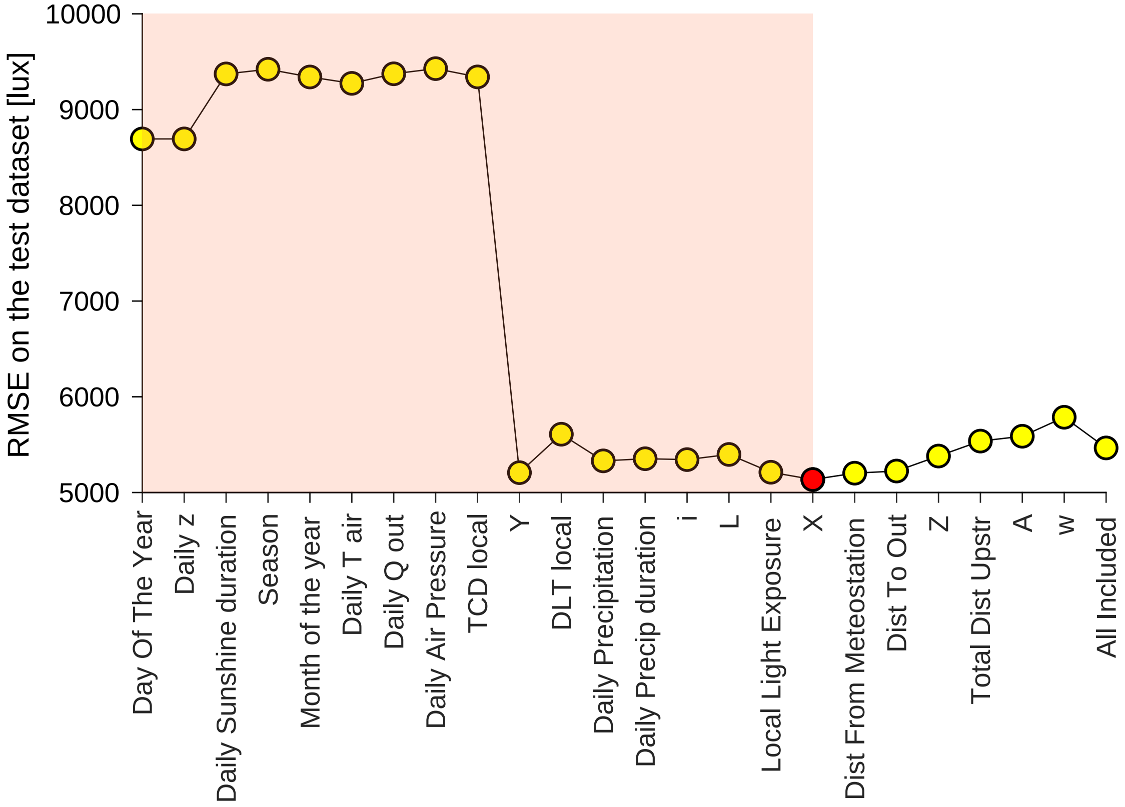

Figure S1: Feature selection for PAR RF trained under setup **S**. Variables have been ranked according to their cumulative OOB importance (sum of the OOB variable importance of the 12 forests trained). Prediction error (RMSE over the 12 sites excluded one a time) is plotted against the subset of features used as learners: all features on the left of each circle are included. Red shaded area represents the feature region that minimizes the prediction error (see Methods section in the main text).

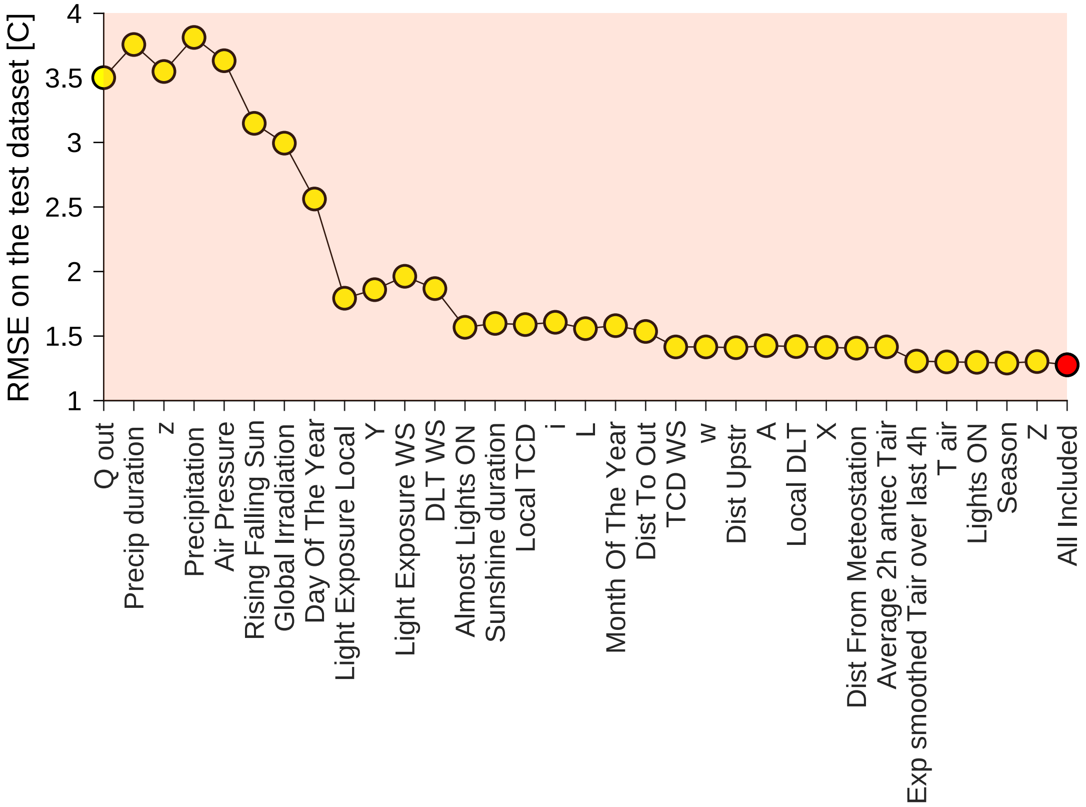

Figure S2: Feature selection for T RF trained under setup **S**. Variables have been ranked according to their cumulative OOB importance (sum of the OOB variable importance of the 12 forests trained). Prediction error (RMSE over the 12 sites excluded one a time) is plotted against the subset of features used as learners: all features on the left of each circle are included. Red shaded area represents the feature region that minimizes the prediction error (see Methods section in the main text).

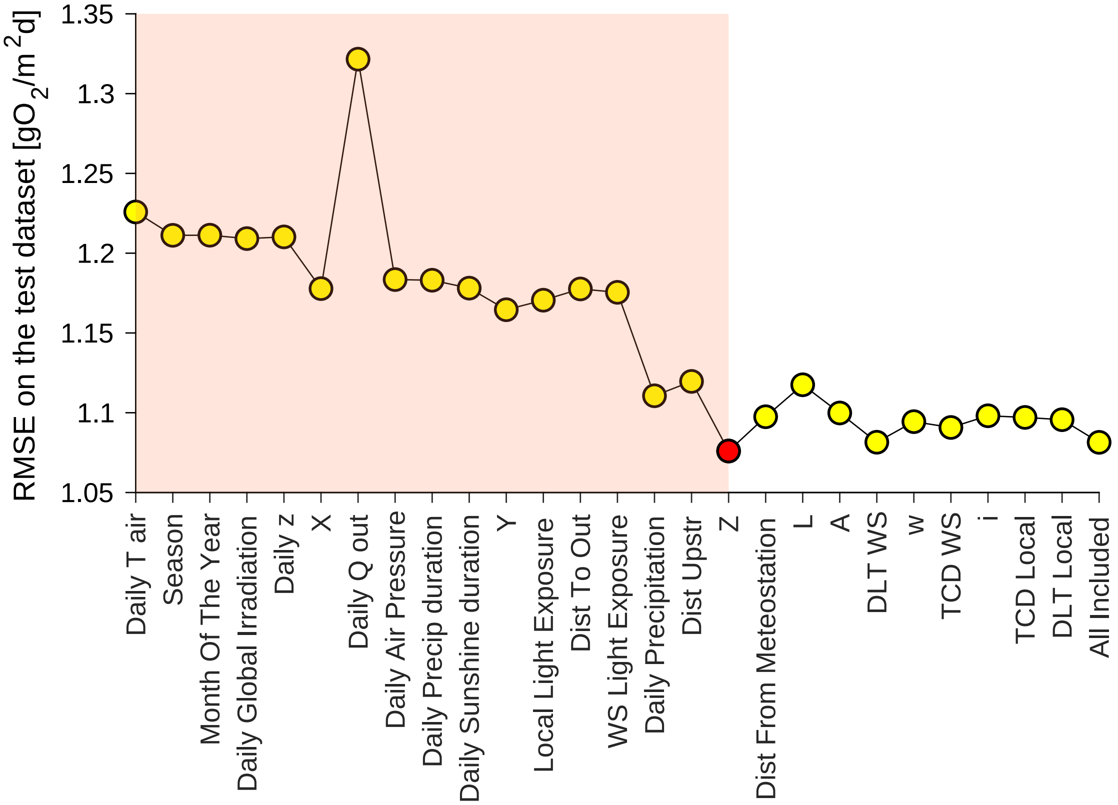

Figure S3: Feature selection for GPP RF trained under setup **S**. Variables have been ranked according to their cumulative OOB importance (sum of the OOB variable importance of the 12 forests trained). Prediction error (RMSE over the 12 sites excluded one a time) is plotted against the subset of features used as learners: all features on the left of each circle are included. Red shaded area represents the feature region that minimizes the prediction error (see Methods section in the main text).

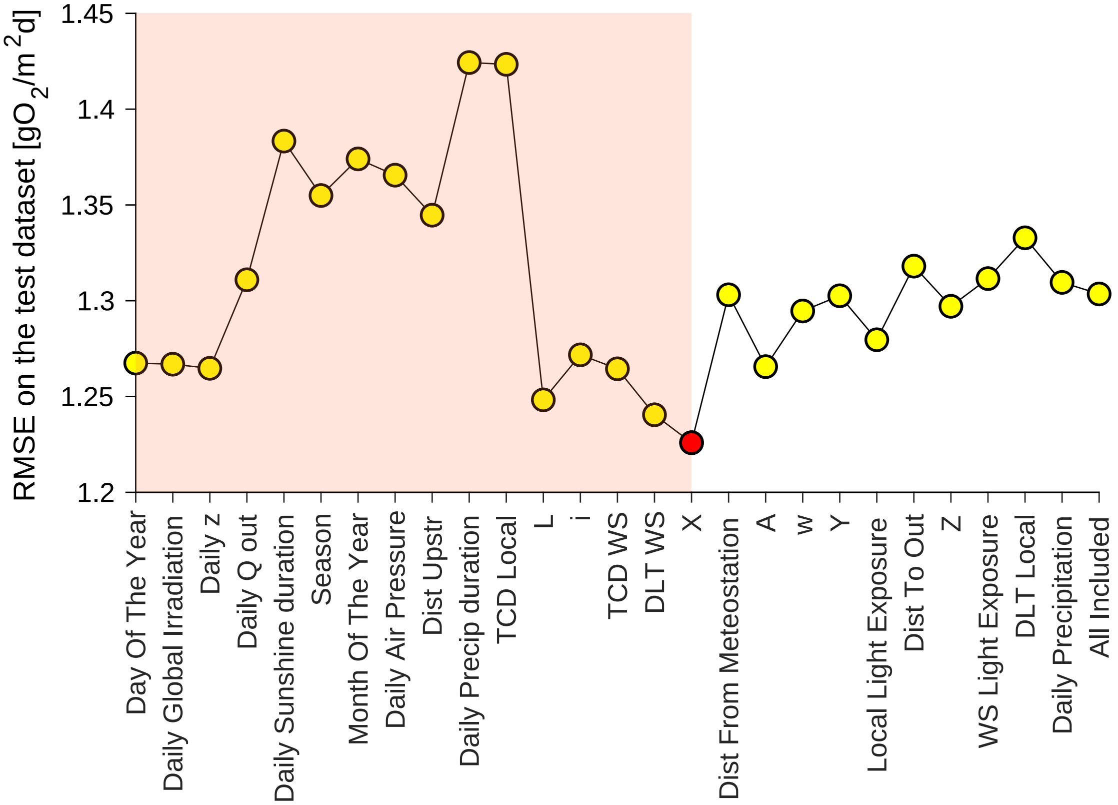

Figure S4: Feature selection for ER RF trained under setup **S**. Variables have been ranked according to their cumulative OOB importance (sum of the OOB variable importance of the 12 forests trained). Prediction error (RMSE over the 12 sites excluded one a time) is plotted against the subset of features used as learners: all features on the left of each circle are included. Red shaded area represents the feature region that minimizes the prediction error (see Methods section in the main text).

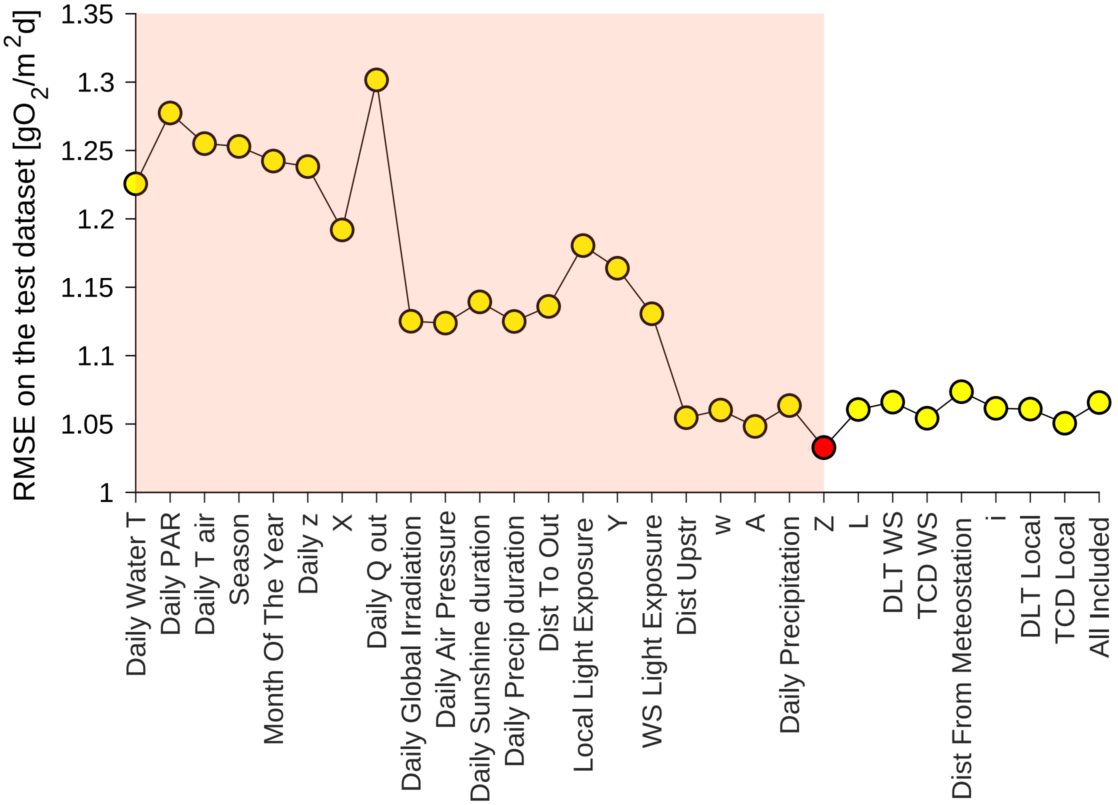

Figure S5: Feature selection for GPP RF including T and PAR predictions as features and trained under setup **S**. Variables have been ranked according to their cumulative OOB importance (sum of the OOB variable importance of the 12 forests trained). Prediction error (RMSE over the 12 sites excluded one a time) is plotted against the subset of features used as learners: all features on the left of each circle are included. Red shaded area represents the feature region that minimizes the prediction error (see Methods section in the main text).

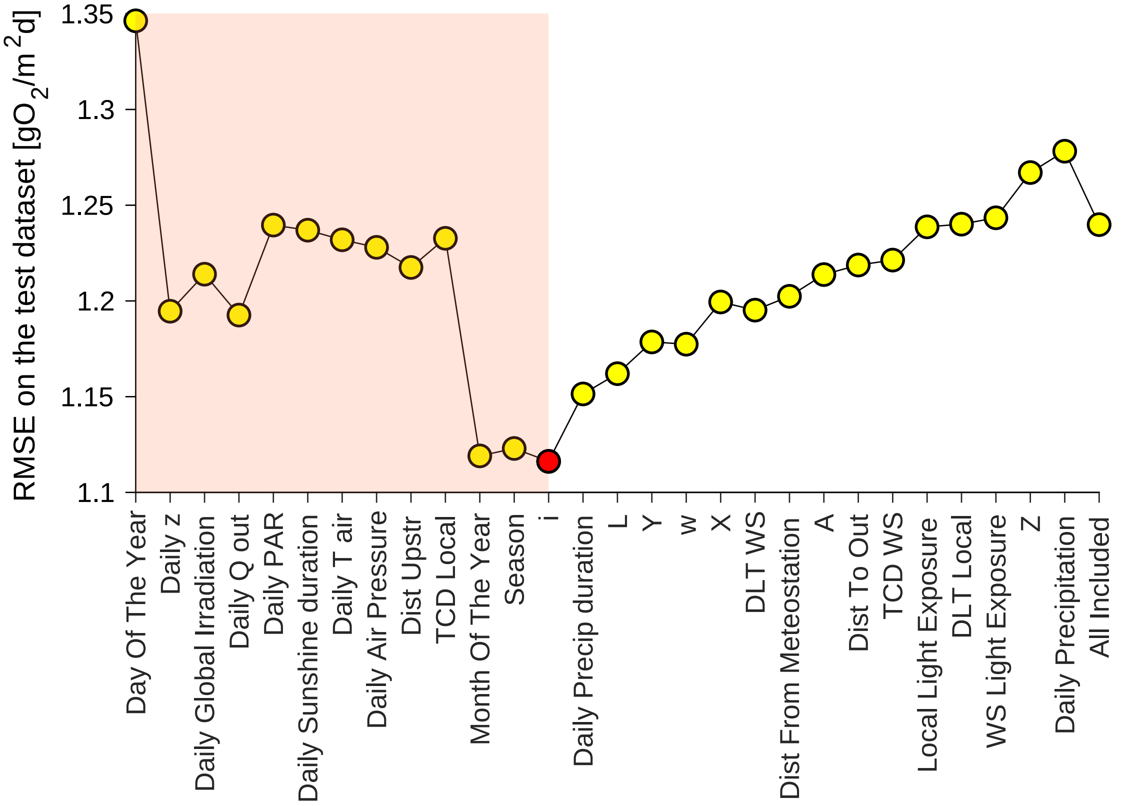

Figure S6: Feature selection for ER RF including T and PAR predictions as features and trained under setup **S**. Variables have been ranked according to their cumulative OOB importance (sum of the OOB variable importance of the 12 forests trained). Prediction error (RMSE over the 12 sites excluded one a time) is plotted against the subset of features used as learners: all features on the left of each circle are included. Red shaded area represents the feature region that minimizes the prediction error (see Methods section in the main text).

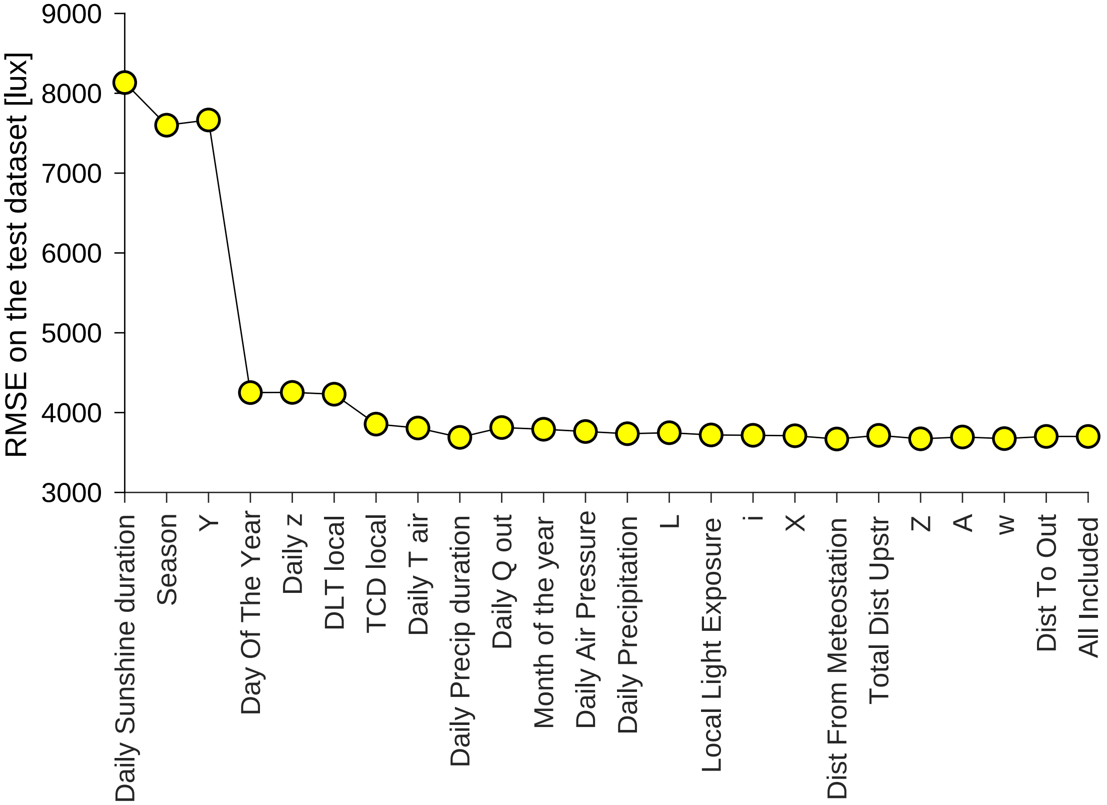

Figure S7: Feature selection for PAR RF trained under setup **T**. Variables have been ranked according to their cumulative OOB importance (sum of the OOB variable importance). Prediction error in the timeseries excluded from training is plotted against the subset of features used as learners: all features on the left of each circle are included (see Methods section in the main text).

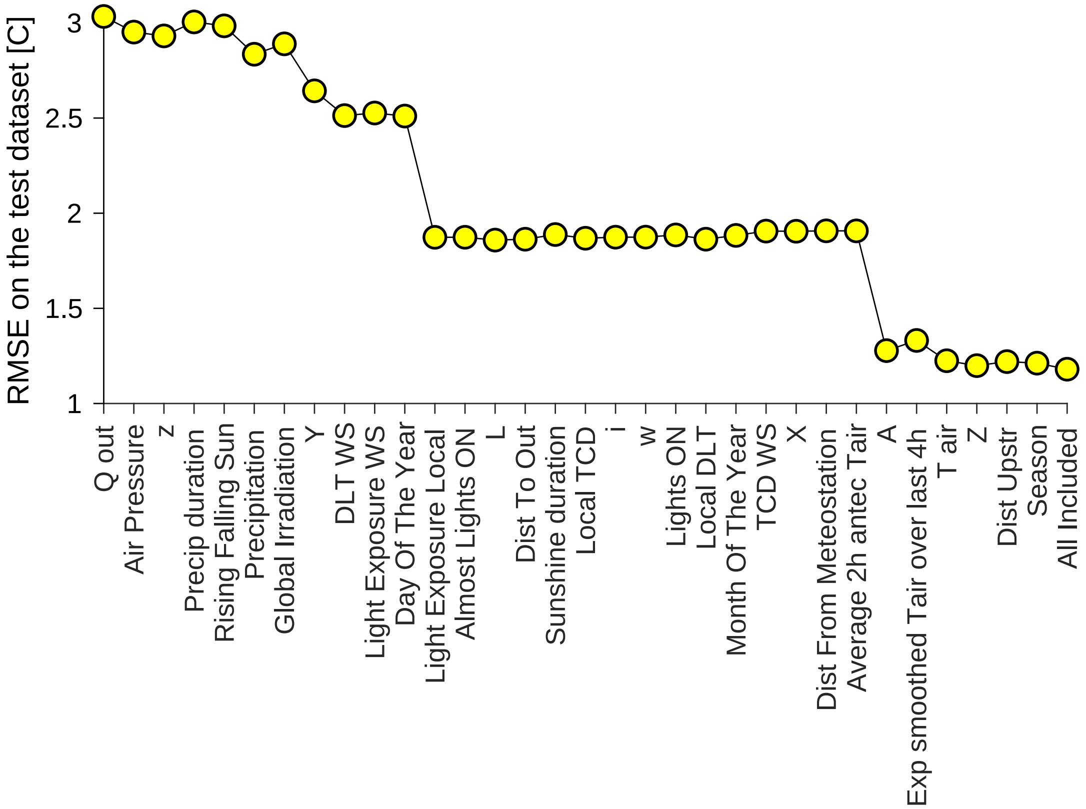

Figure S8: Feature selection for T RF trained under setup **T**. Variables have been ranked according to their cumulative OOB importance (sum of the OOB variable importance). Prediction error in the timeseries excluded from training is plotted against the subset of features used as learners: all features on the left of each circle are included (see Methods section in the main text).

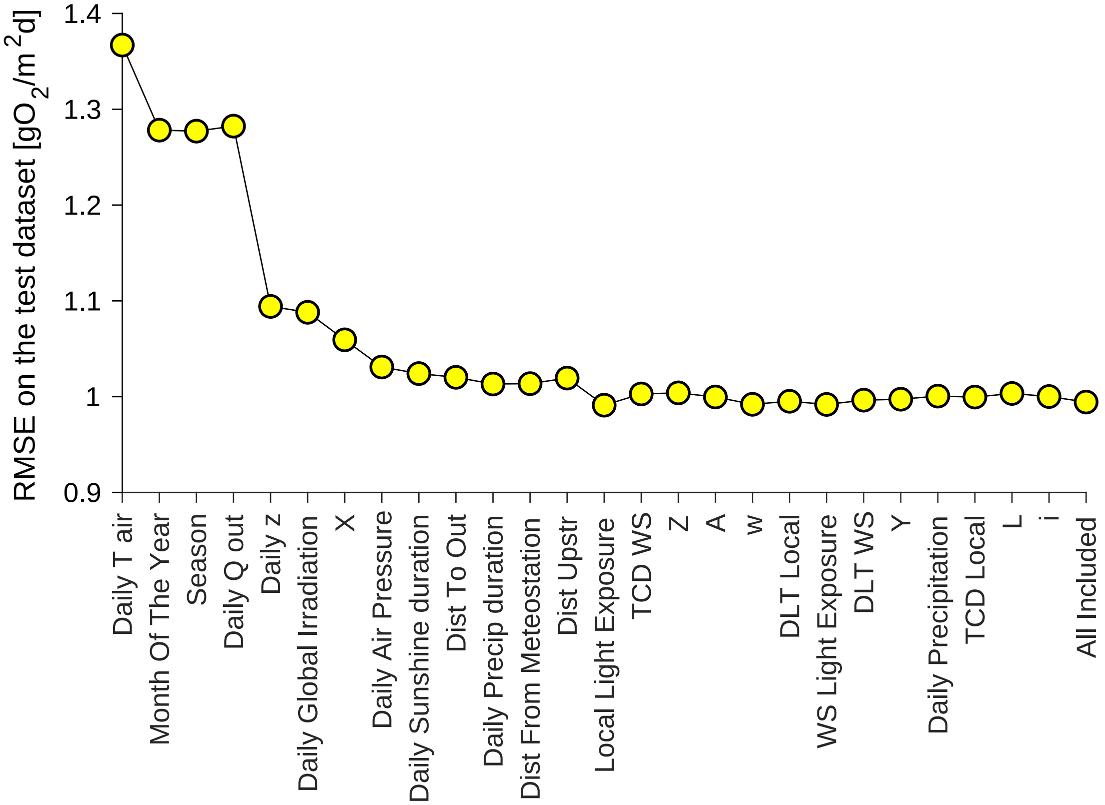

Figure S9: Feature selection for GPP RF trained under setup **T**. Variables have been ranked according to their cumulative OOB importance (sum of the OOB variable importance). Prediction error in the timeseries excluded from training is plotted against the subset of features used as learners: all features on the left of each circle are included (see Methods section in the main text).

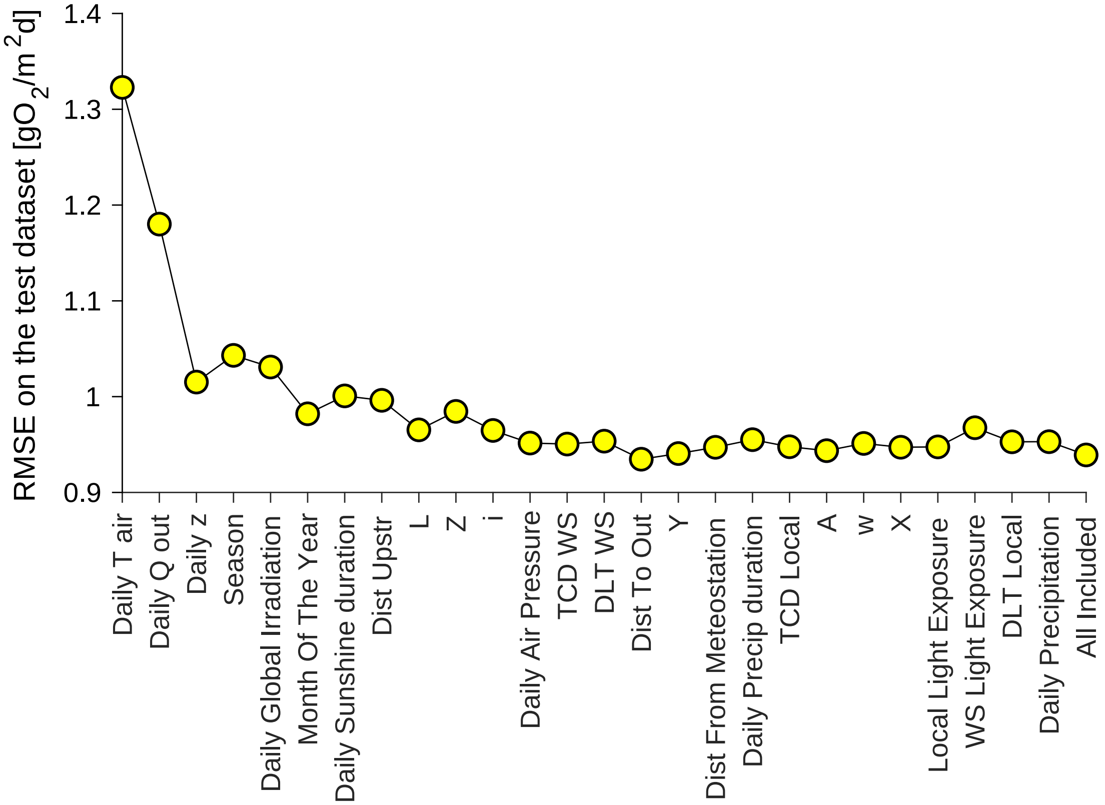

Figure S10: Feature selection for ER RF trained under setup **T**. Variables have been ranked according to their cumulative OOB importance (sum of the OOB variable importance). Prediction error in the timeseries excluded from training is plotted against the subset of features used as learners: all features on the left of each circle are included (see Methods section in the main text).

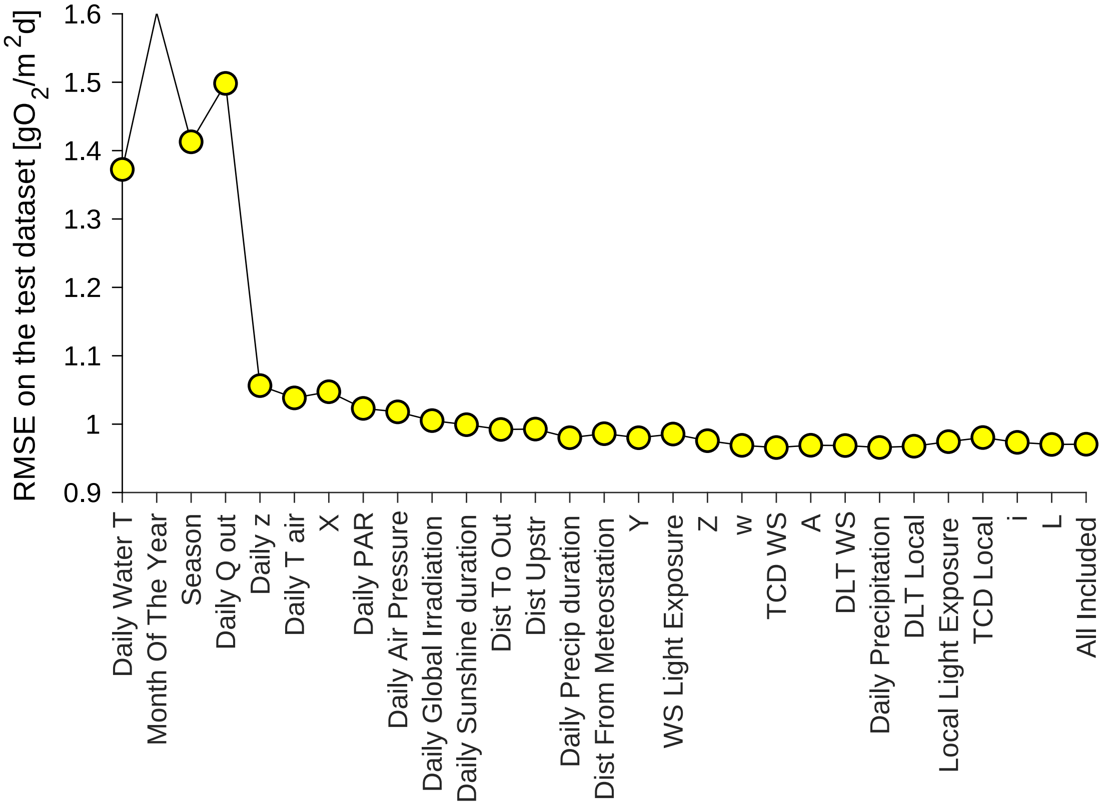

Figure S11: Feature selection for GPP RF including T and PAR predictions as features and trained under setup **T**. Variables have been ranked according to their cumulative OOB importance (sum of the OOB variable importance). Prediction error in the timeseries excluded from training is plotted against the subset of features used as learners: all features on the left of each circle are included (see Methods section in the main text).

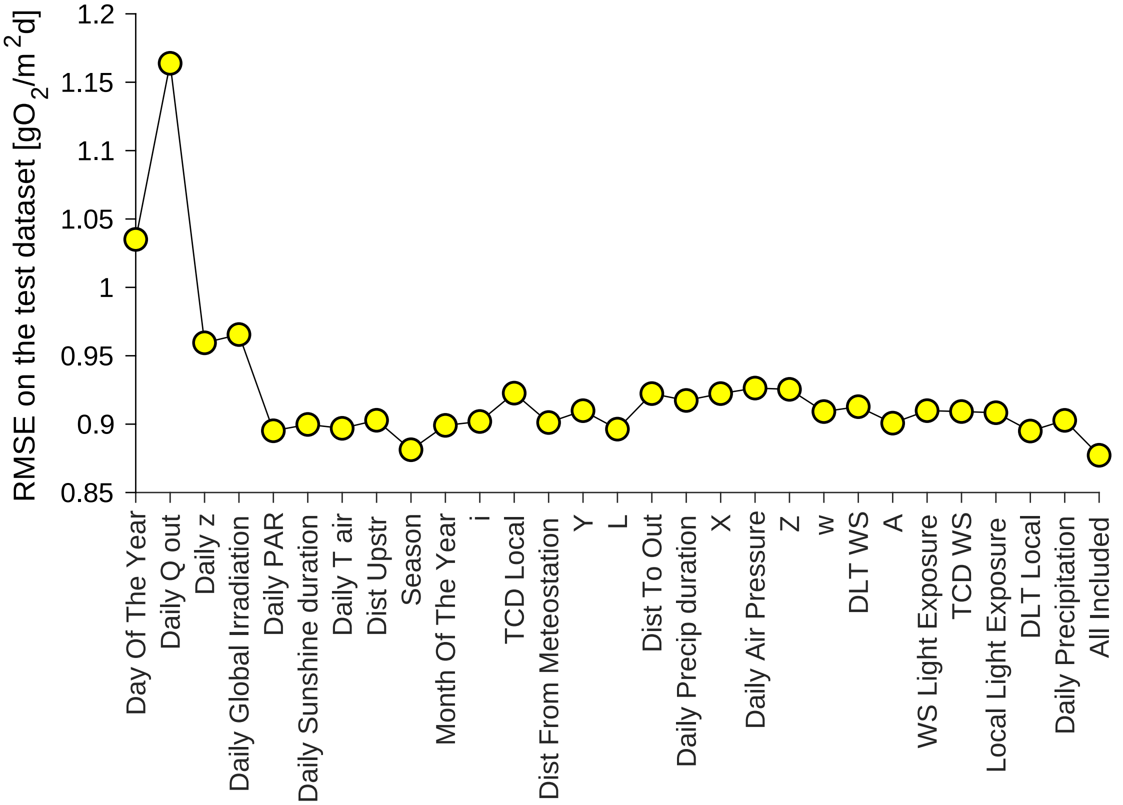

Figure S12: Feature selection for ER RF including T and PAR predictions as features and trained under setup **T**. Variables have been ranked according to their cumulative OOB importance (sum of the OOB variable importance). Prediction error in the timeseries excluded from training is plotted against the subset of features used as learners: all features on the left of each circle are included (see Methods section in the main text).

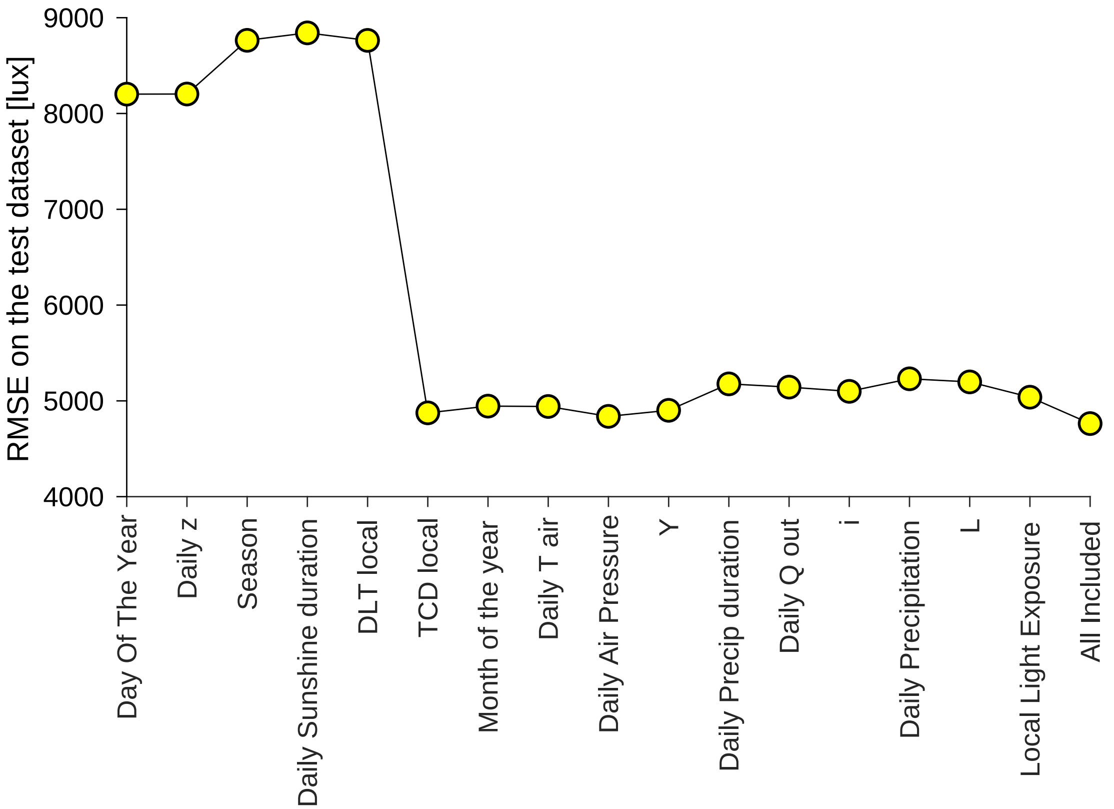

Figure S13: PAR RF, re-training under setup **S** using the best predictors selected (Figure S1). Overfit check. Symbols as in Figure S1.

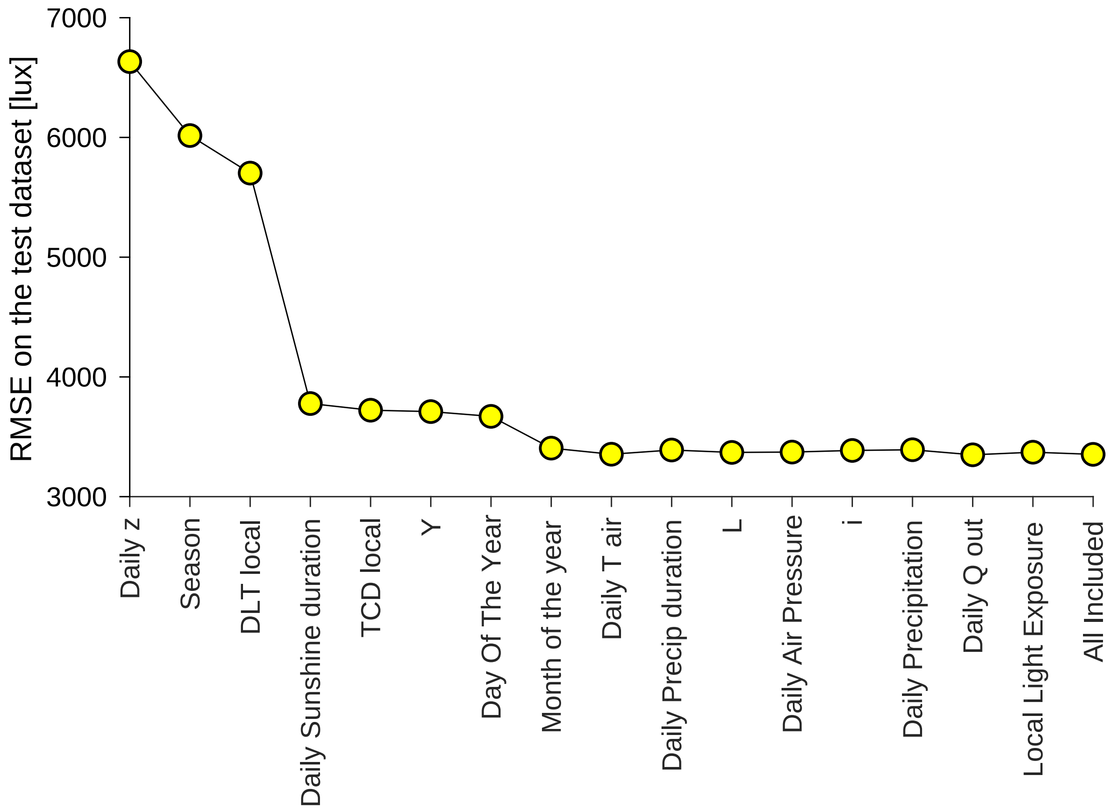

Figure S14: PAR RF, re-training under setup **T** but using only the best predictors selected under training **S** (Figure S1). Overfit check. Symbols as in Figure S1.

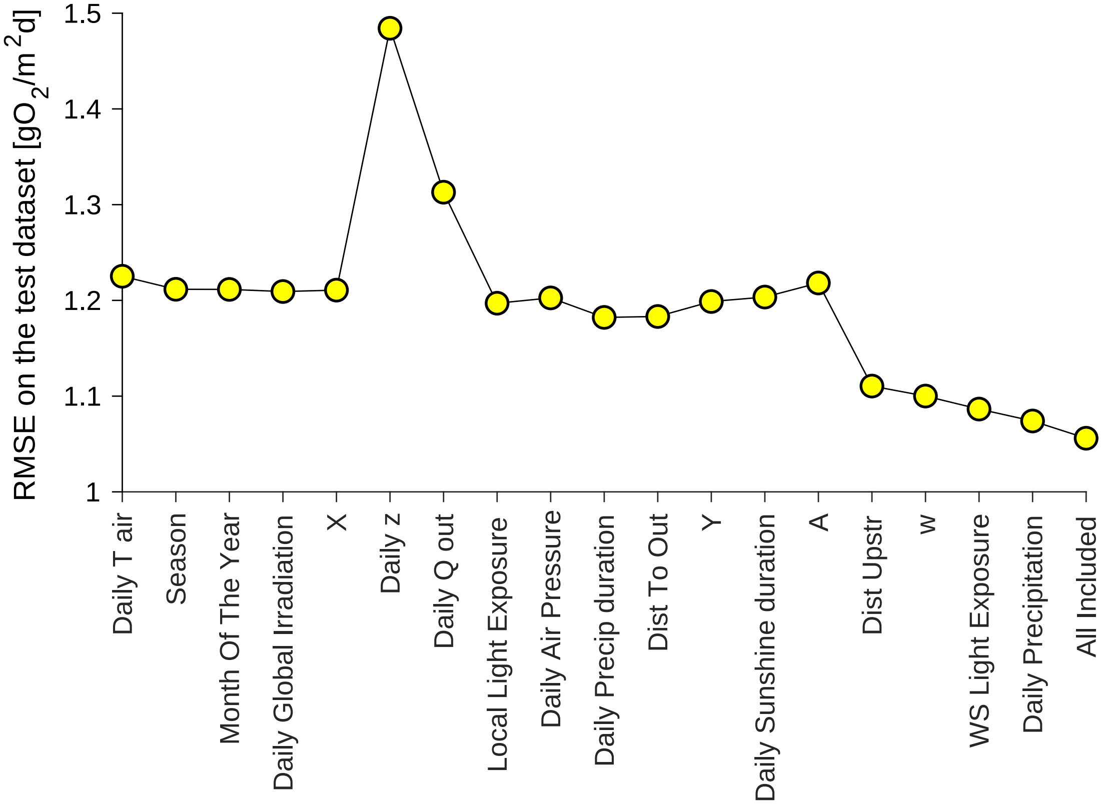

Figure S15: GPP RF, re-training under setup **S** using the best predictors selected (Figure S3). Overfit check. Symbols as in Figure S3.

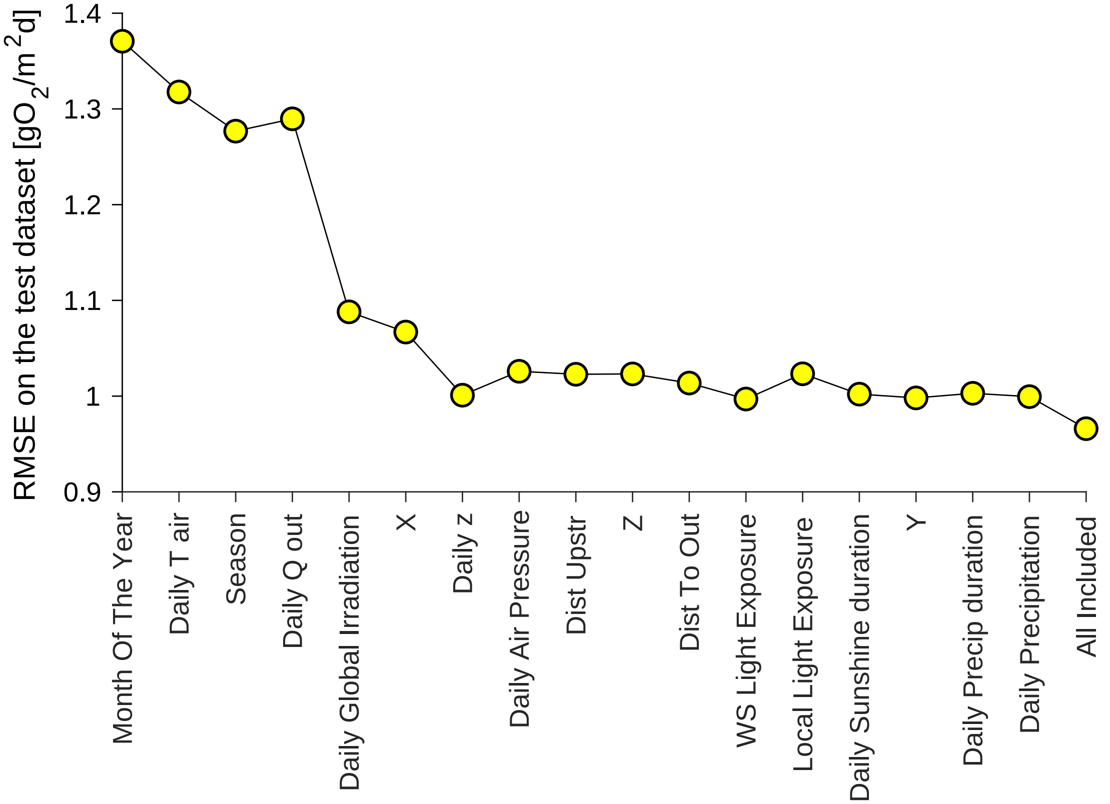

Figure S16: GPP RF, re-training under setup **T** but using only the best predictors selected under training **S** (Figure S3). Overfit check. Symbols as in Figure S3.

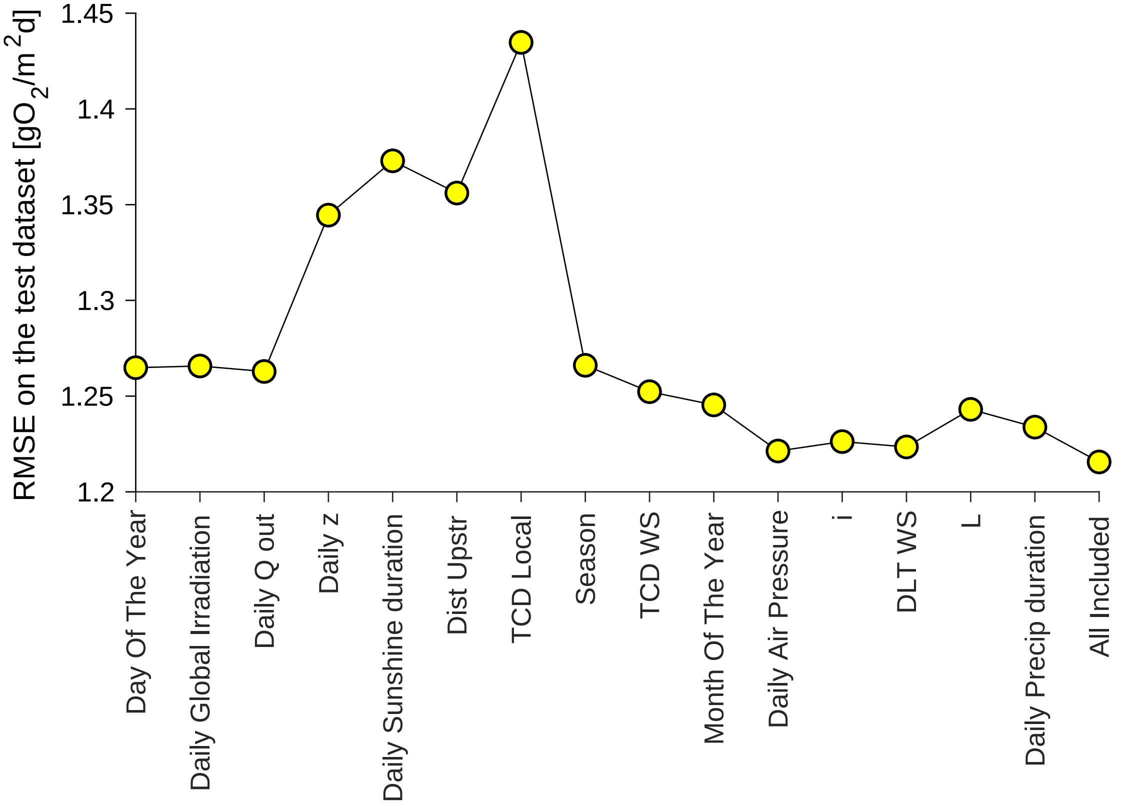

Figure S17: ER RF, re-training under setup **S** using the best predictors selected (Figure S4). Overfit check. Symbols as in Figure S4.

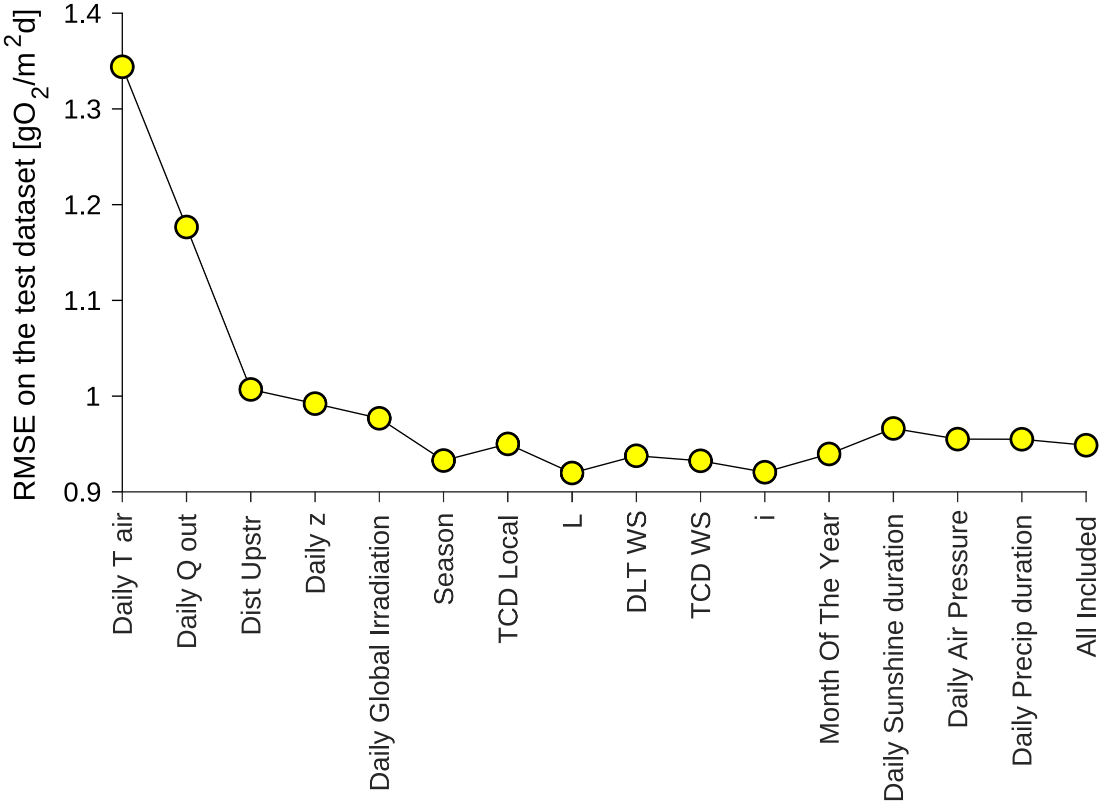

Figure S18: ER RF, re-training under setup **T** but using only the best predictors selected under training **S** (Figure S4). Overfit check. Symbols as in Figure S4.

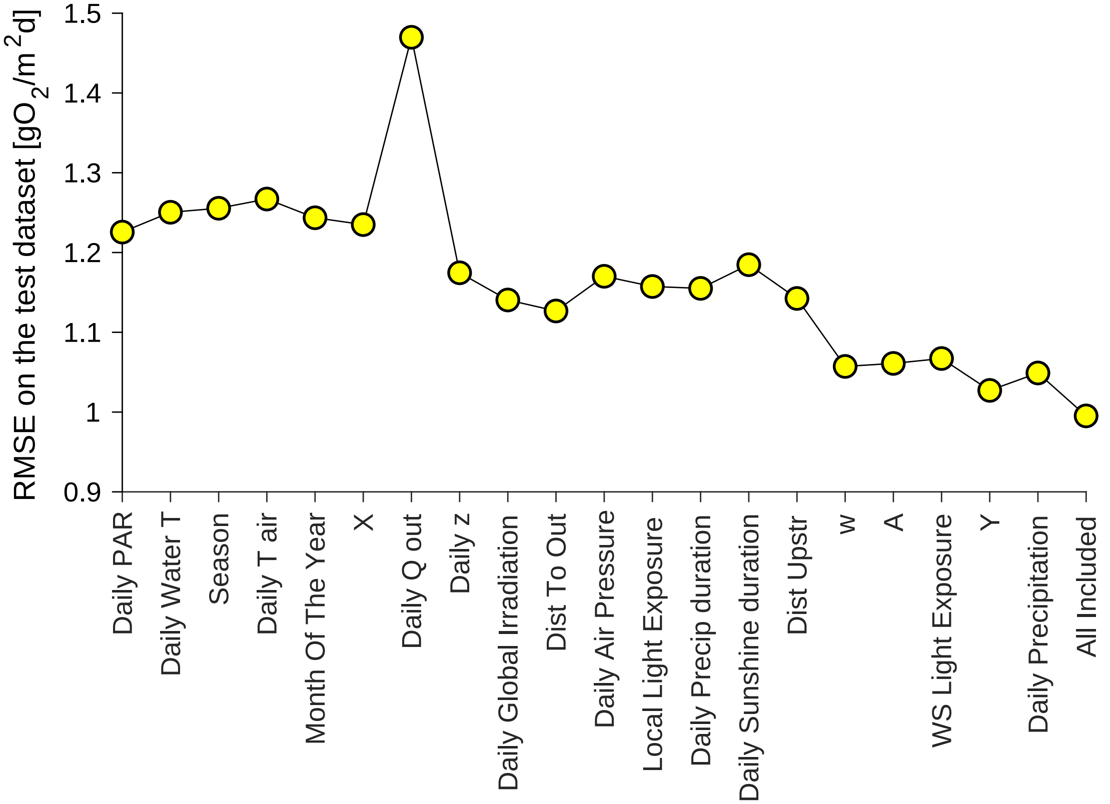

Figure S19: GPP RF including T and PAR predictions as features, re-training under setup **S** using the best predictors selected (Figure S5). Overfit check. Symbols as in Figure S5.

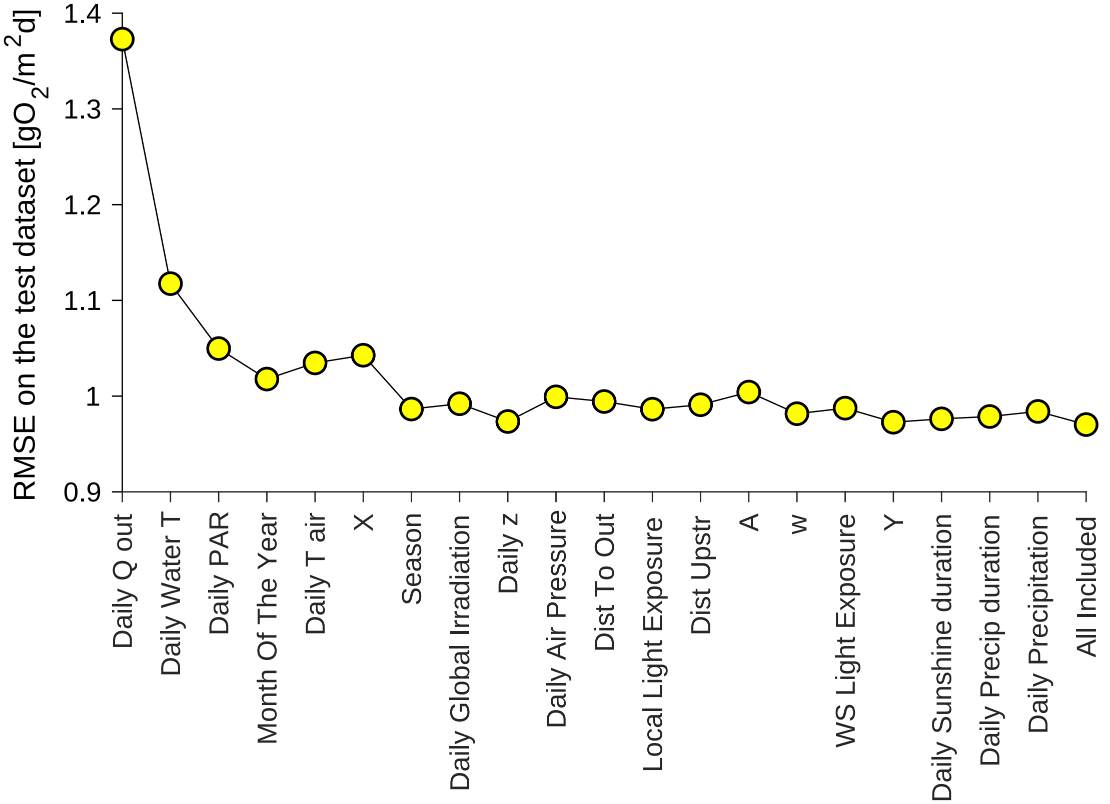

Figure S20: GPP RF including T and PAR predictions as features, re-training under setup **T** but using only the best predictors selected under training **S** (Figure S5). Overfit check. Symbols as in Figure S5.

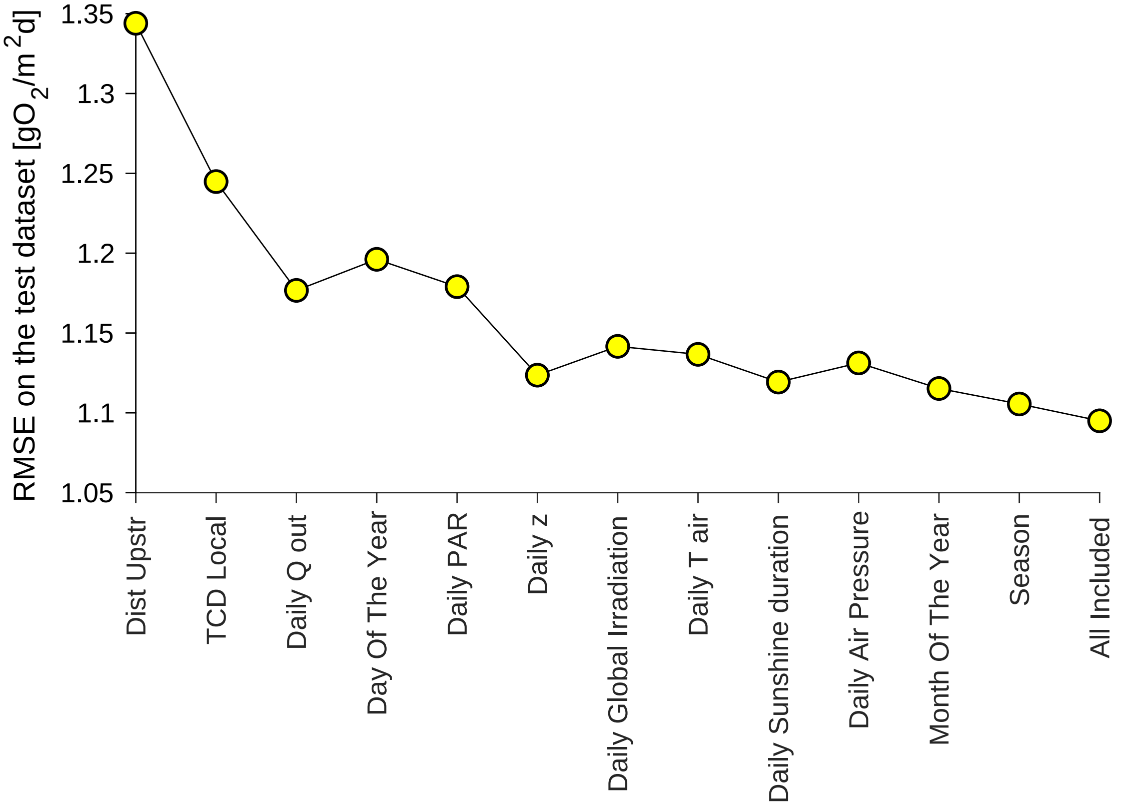

Figure S21: ER RF including T and PAR predictions as features, re-training under setup **S** using the best predictors selected (Figure S6). Overfit check. Symbols as in Figure S6.

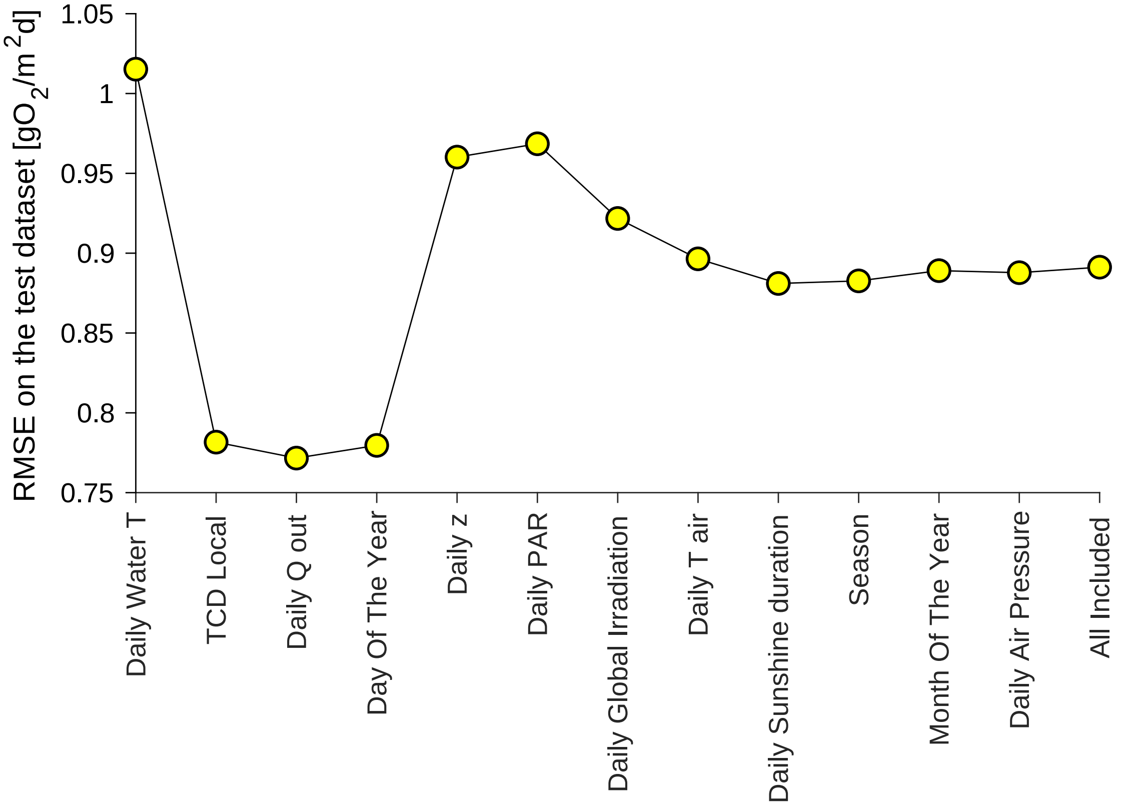

Figure S22: ER RF including T and PAR predictions as features, re-training under setup **T** but using only the best predictors selected under training **S** (Figure S6). Overfit check. Symbols as in Figure S6.

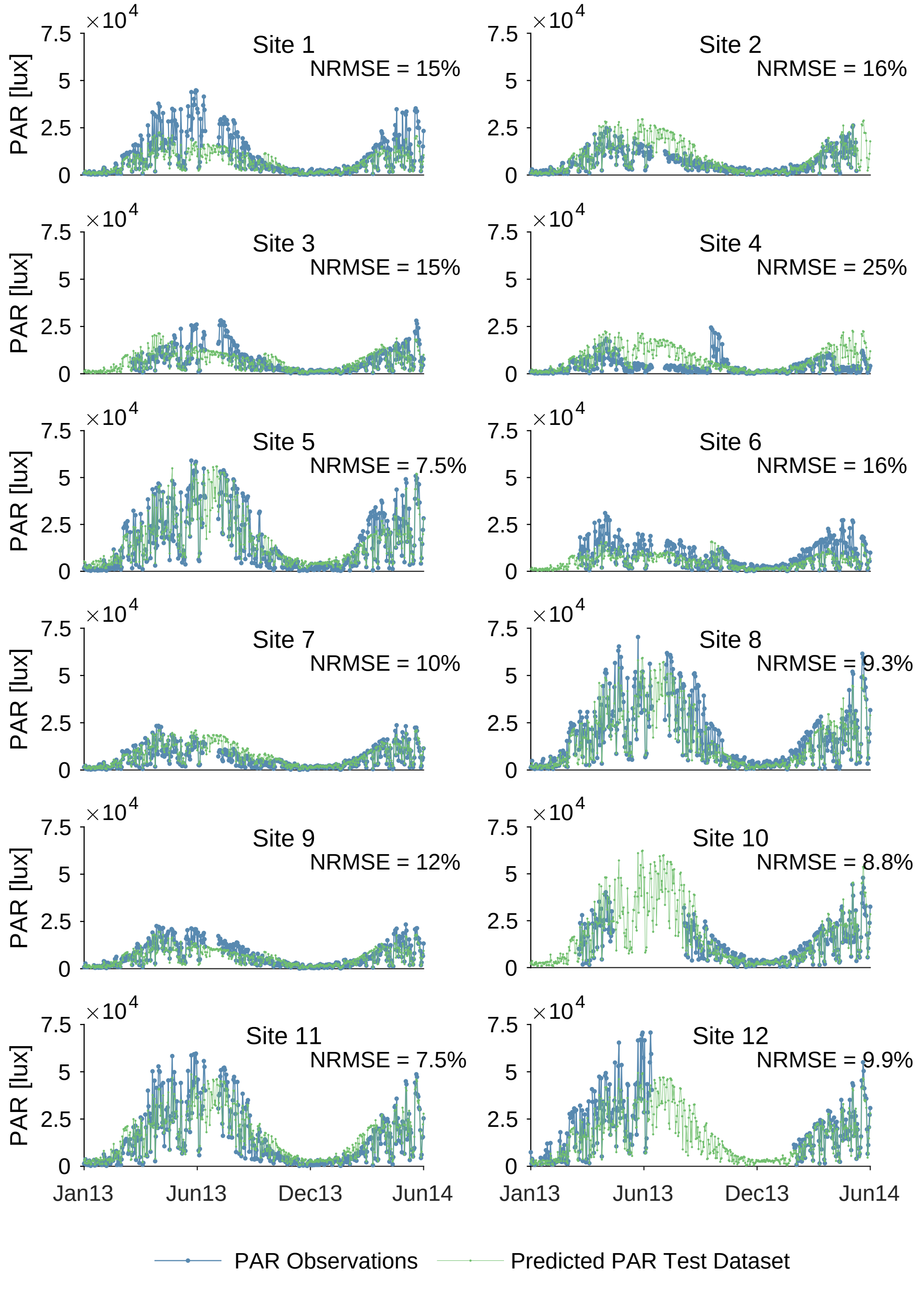

Figure S23: Model output of PAR RF trained under setup **S**. Each subplot depicts one of the twelve sites and shows measured (blue dotted line) against predicted signals (green dotted line). Each predicted time-series refers to the data-set not used in training, i.e. it has been derived using the RF (one of the twelve trained using the best predictors, see Table S1) that has never seen that specific site during training.

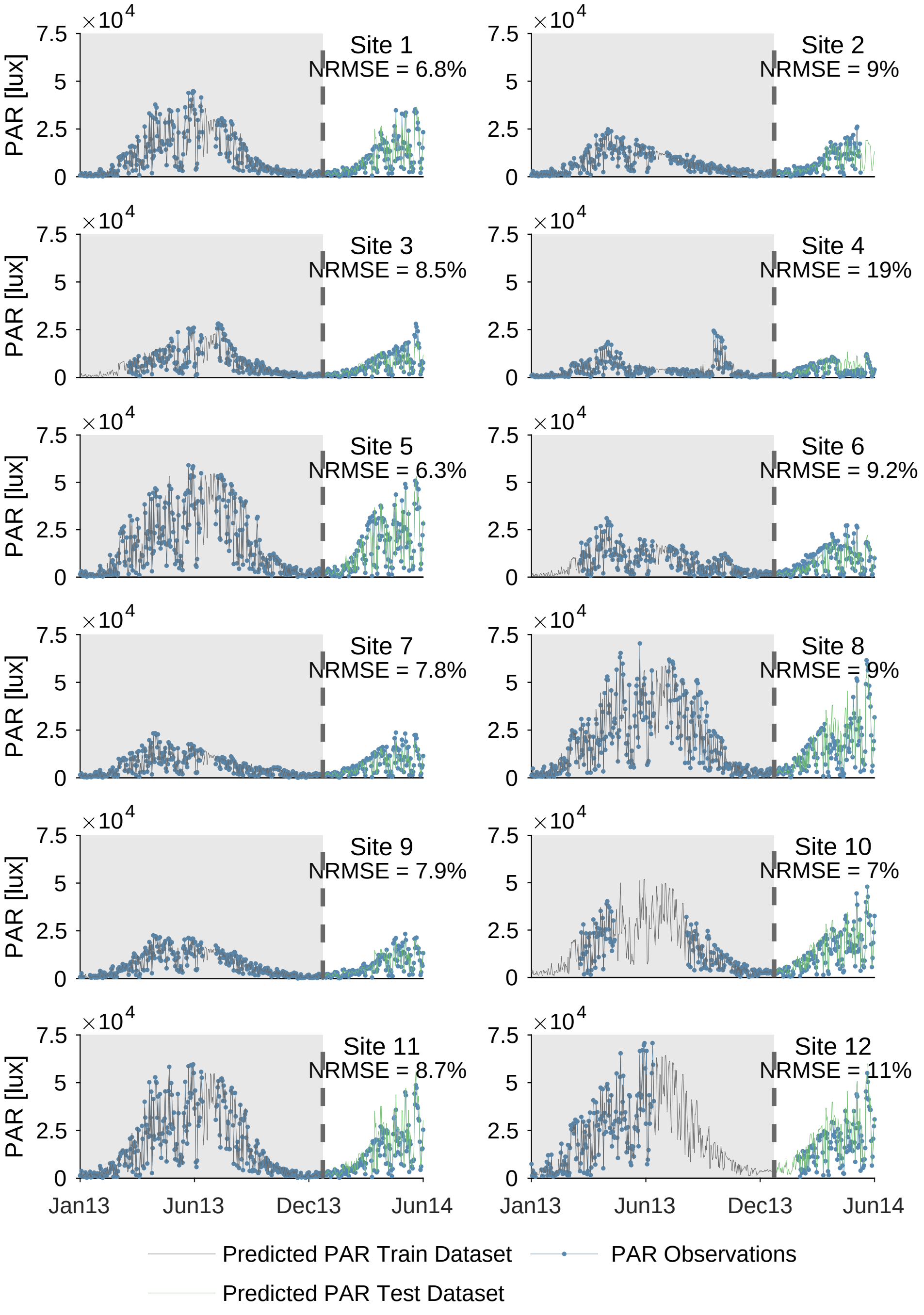

Figure S24: Model output of PAR RF trained under setup **T**and using the best predictors selected under training **S** (see Table S1). Each subplot depicts one of the twelve sites and shows measured (blue dotted line) against predicted signals. The test data-set corresponds to the last six months of the whole time series (green dotted line). Predictions before Jan 2014 (gray dotted line) have not been considered for the error estimation as they have been used when training the ensemble algorithm.

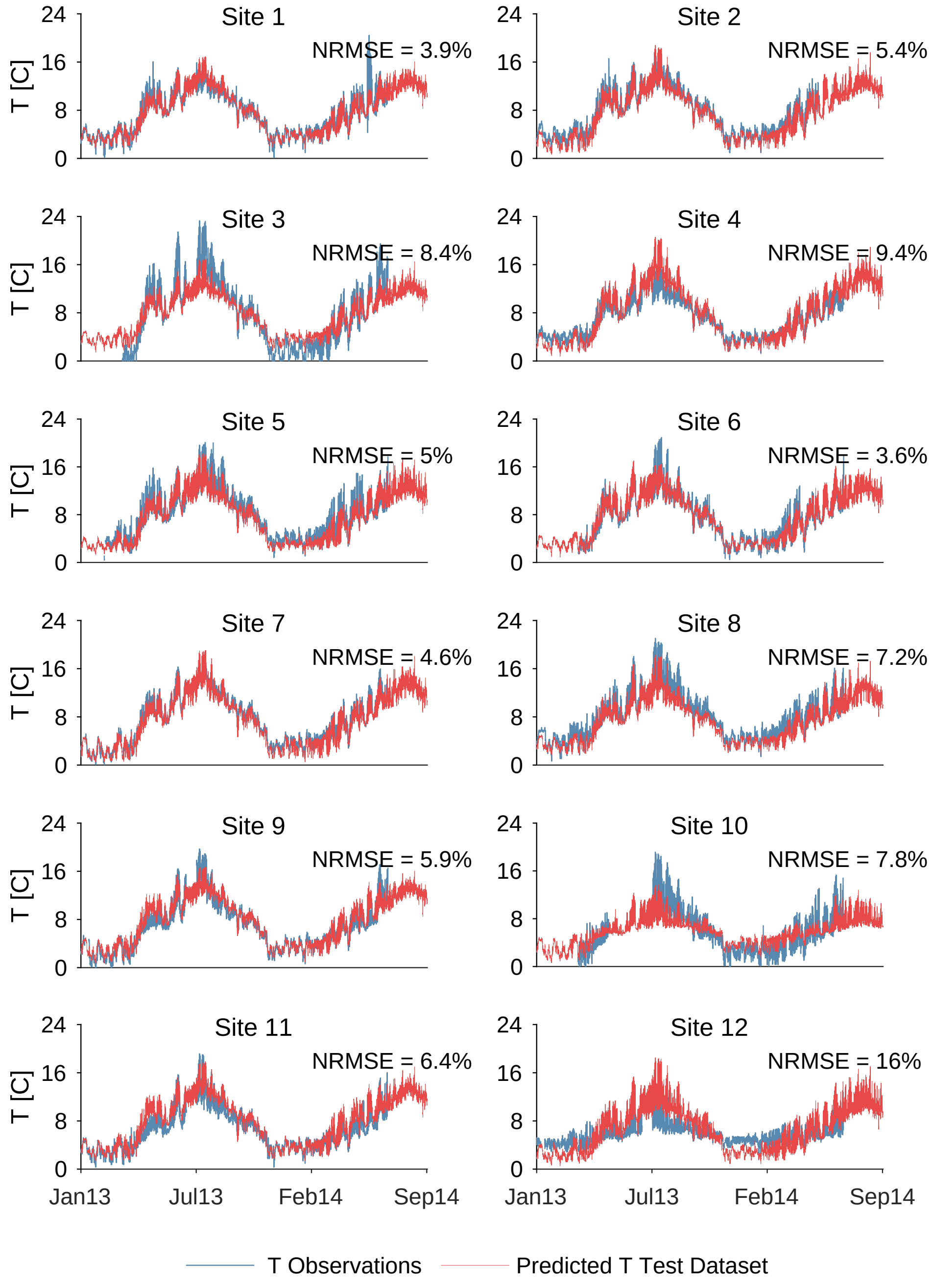

Figure S25: Model output of T RF trained under setup **S**. Each subplot depicts one of the twelve sites and shows measured (blue dotted line) against predicted signals (red dotted line). Each predicted time-series refers to the data-set not used in training, i.e. it has been derived using the RF (one of the twelve trained using the best predictors, see Table S1) that has never seen that specific site during training.

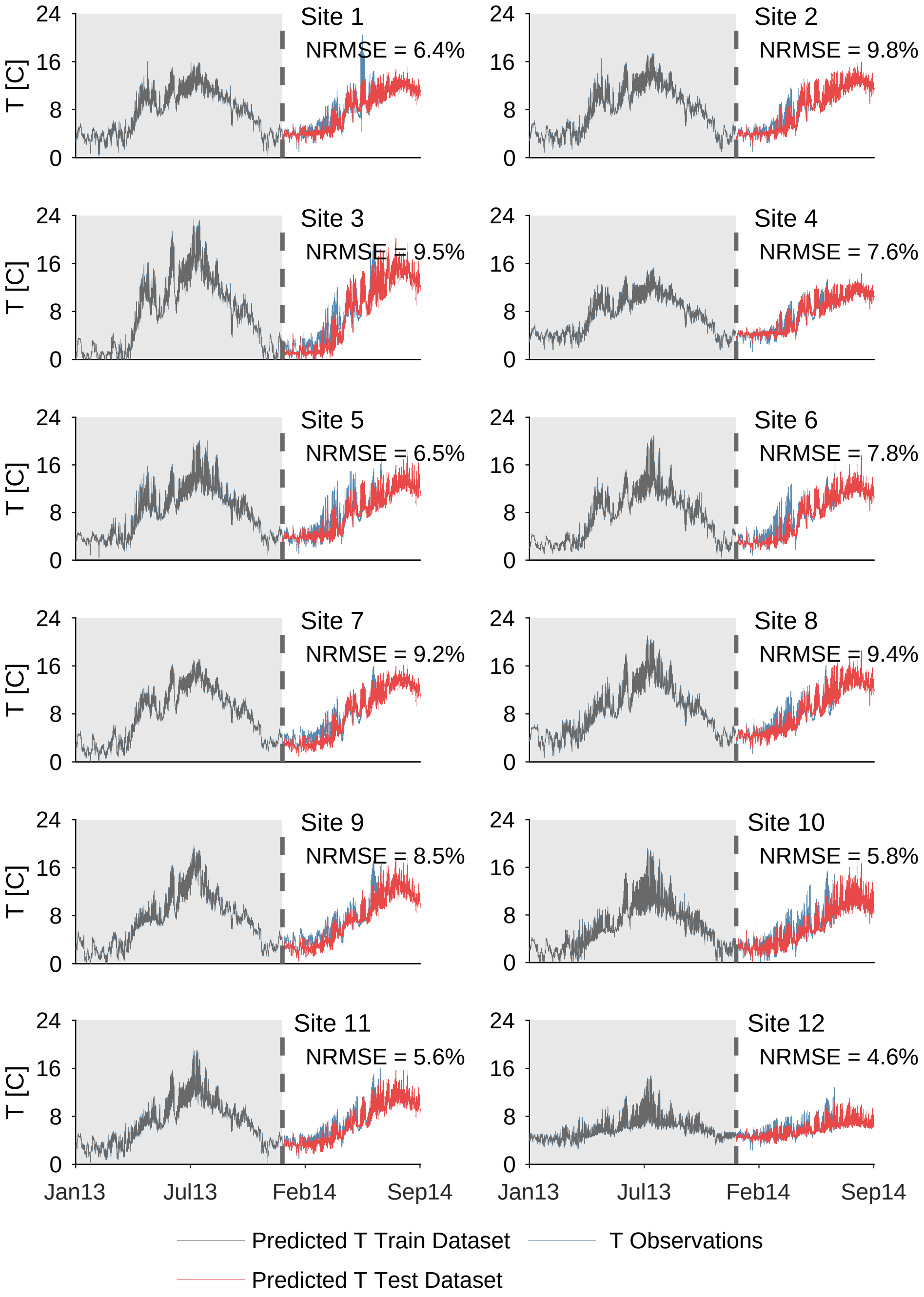

Figure S26: Model output of T RF trained under setup **T** and using the best predictors selected under training **S** (see Table S1). Each subplot depicts one of the twelve sites and shows measured (blue dotted line) against predicted signals. The test data-set corresponds to the last six months of the whole time series (red dotted line). Predictions before Jan 2014 (gray dotted line) have not been considered for the error estimation as they have been used when training the ensemble algorithm.

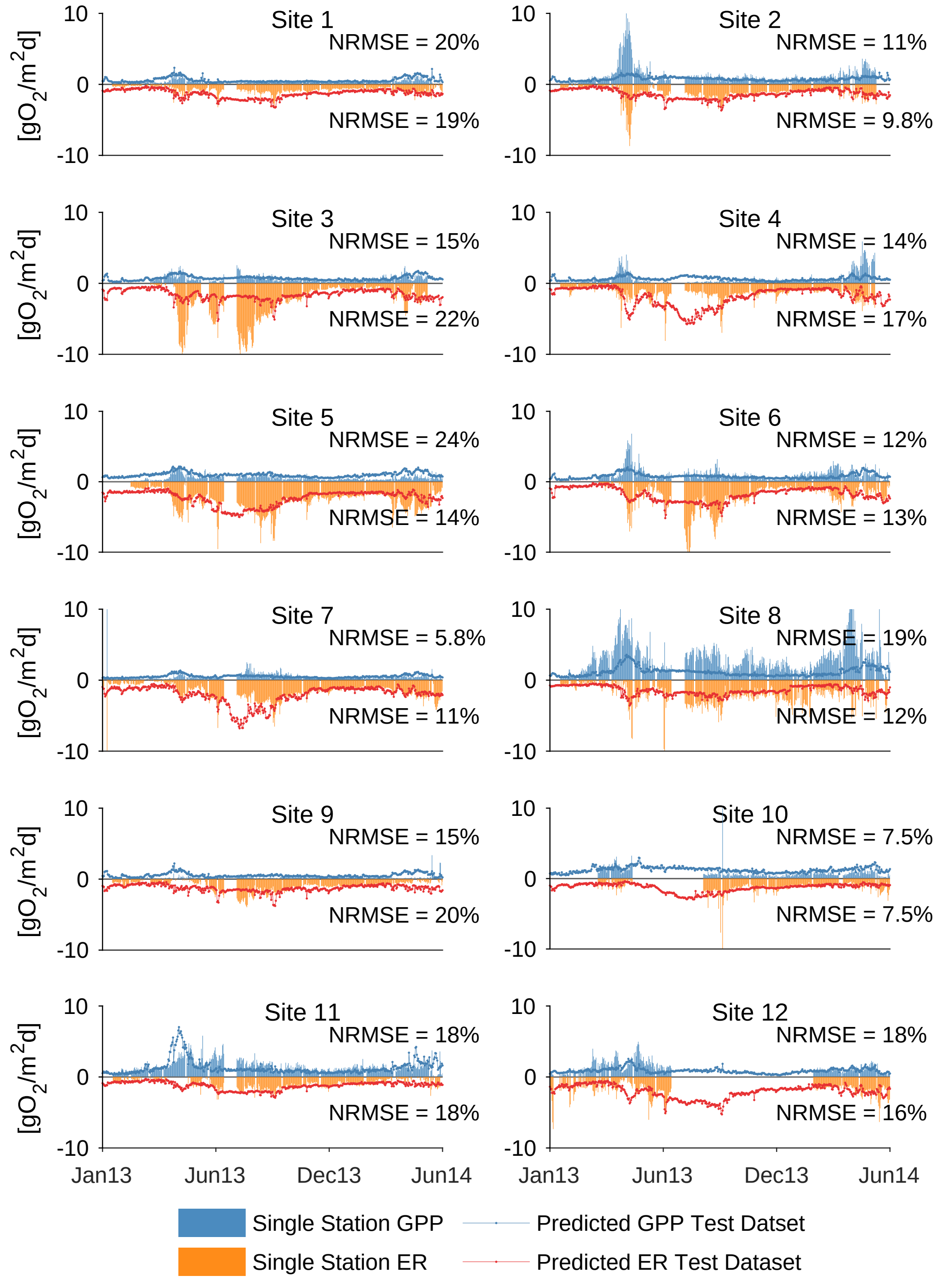

Figure S27: Model output of GPP and ER RF trained under setup **S**. Each subplot depicts one of the twelve sites and shows estimated fluxes using the single station approach (respectively blue bars for GPP and orange for ER) against predicted signals (blue dotted line for GPP and red for ER). Each predicted time-series refers to the data-set not used in training, i.e. it has been derived using the RF (one of the twelve trained using the best predictors, see Table S1) that has never seen that specific site during training.

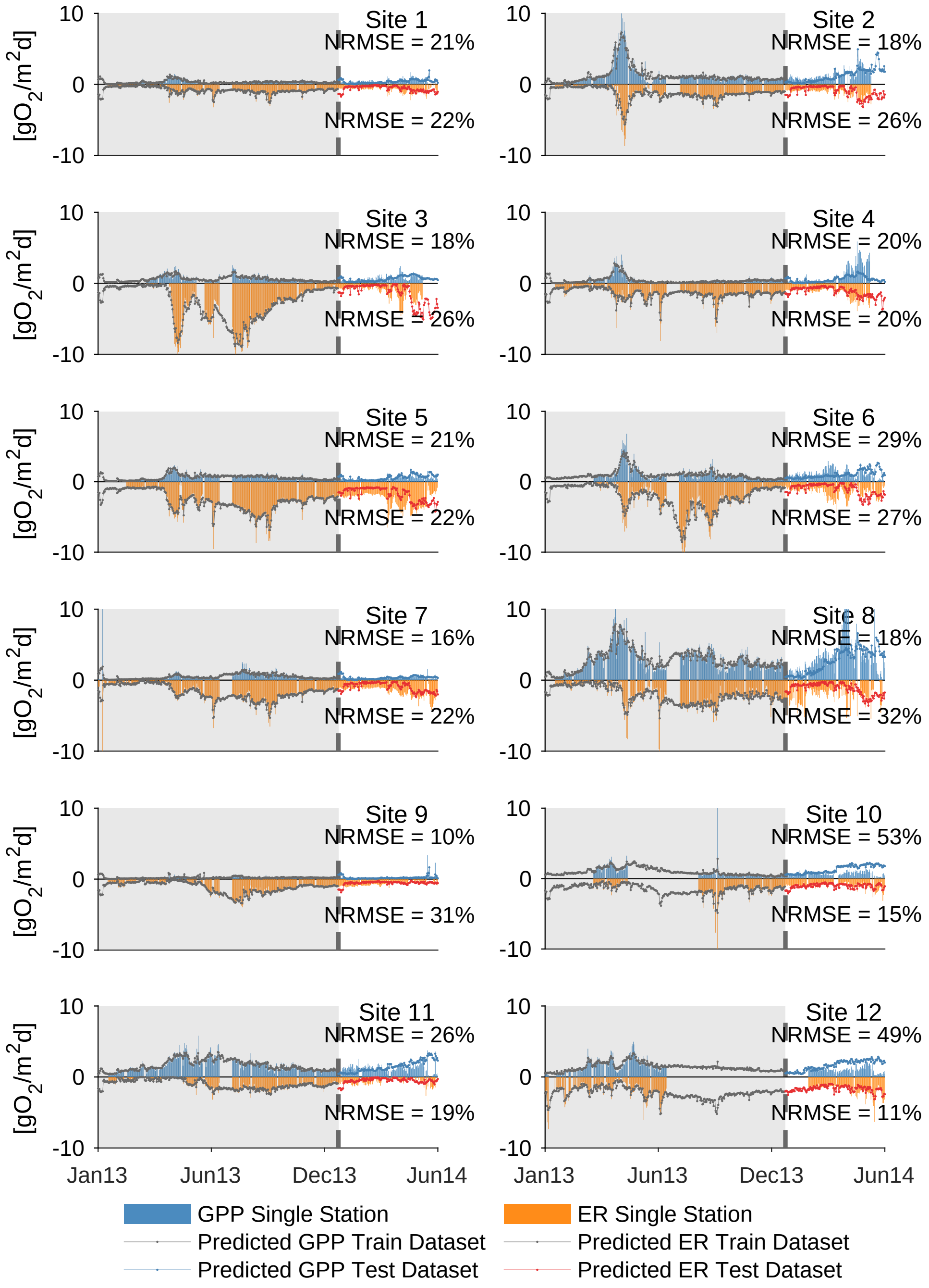

Figure S28: Model output of GPP and ER RF trained under setup **T** and using the best predictors selected under training **S** (see Table S1). Each subplot depicts one of the twelve sites and shows measured (respectively blue bars for GPP and orange for ER) against predicted signals. The test data-set corresponds to the last six months of the whole time series (blue dotted line for GPP and red for ER). Predictions before Jan 2014 (gray dotted line) have not been considered for the error estimation as they have been used when training the ensemble algorithm.

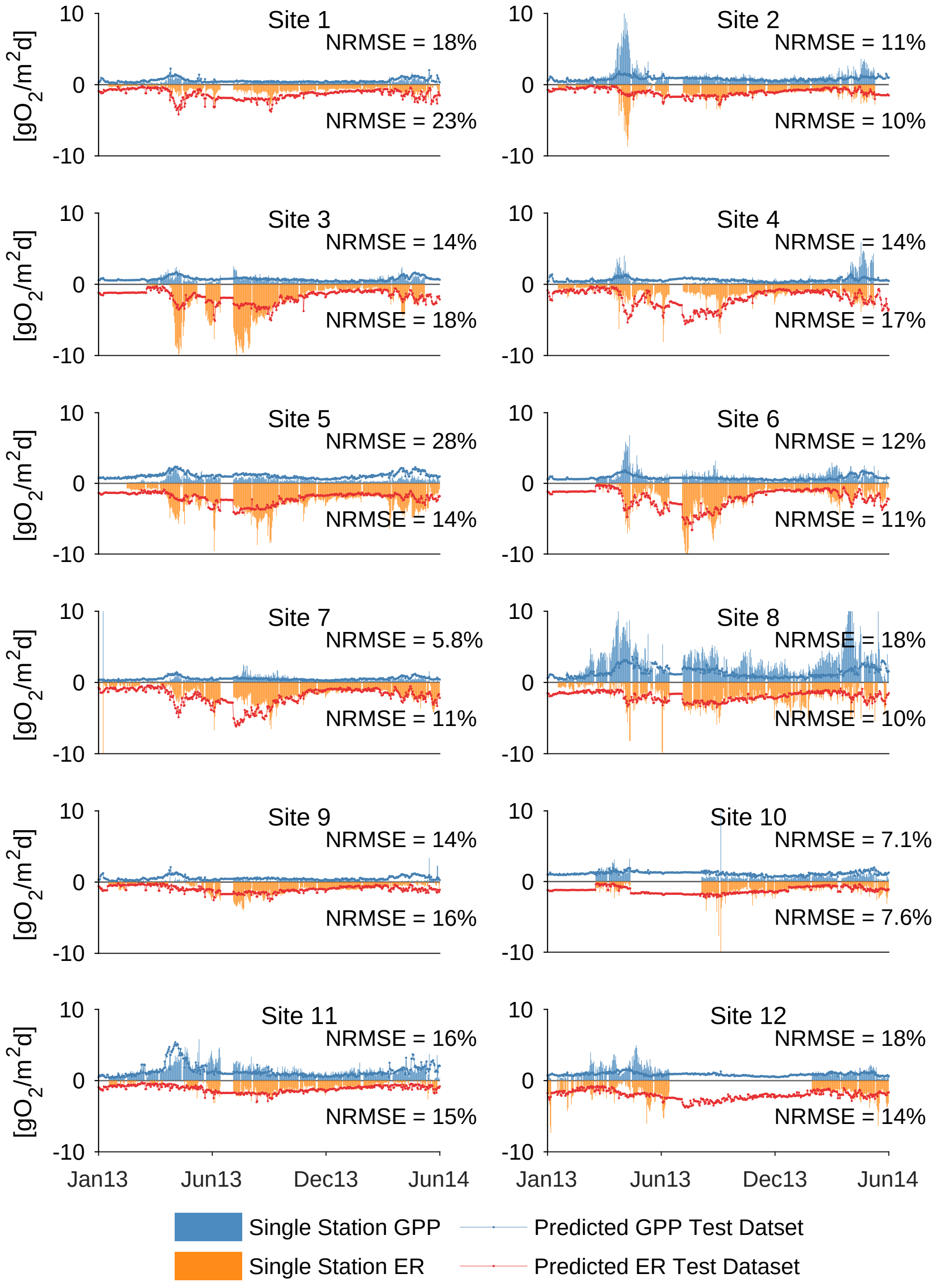

Figure S29: Model output of GPP and ER RF trained under setup **S** and including PAR and stream water T extrapolations as features. Each subplot depicts one of the twelve sites and shows estimated fluxes using the single station approach (respectively blue bars for GPP and orange for ER) against predicted signals (blue dotted line for GPP and red for ER). Each predicted time-series refers to the data-set not used in training, i.e. it has been derived using the RF (one of the twelve trained using the best predictors, see Table S1) that has never seen that specific site during training.

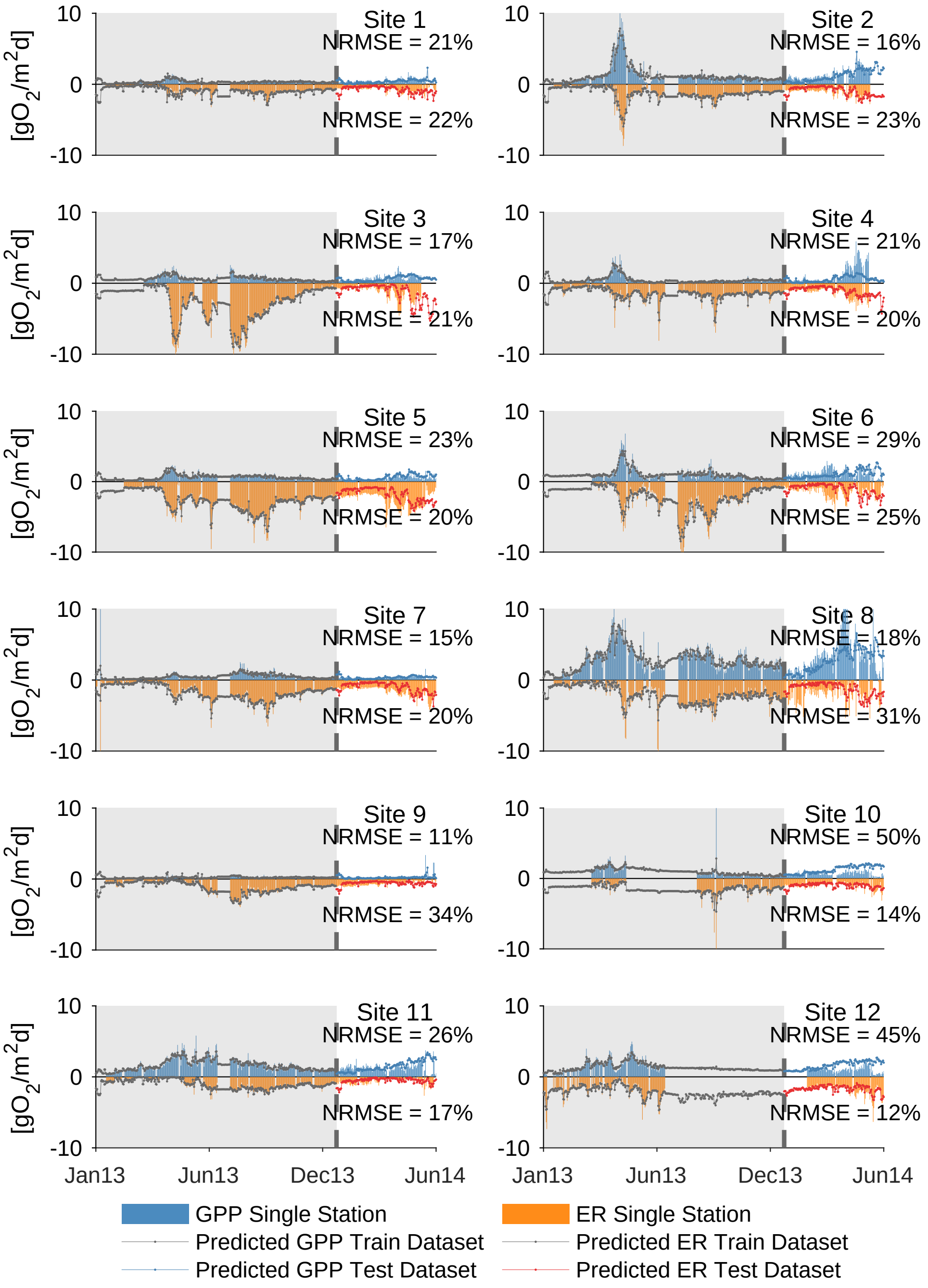

Figure S30: Model output of GPP and ER RF trained under setup **T**, including PAR and stream water T extrapolations as features, and using the best predictors selected under training **S** (see Table S1). Each subplot depicts one of the twelve sites and shows measured (respectively blue bars for GPP and orange for ER) against predicted signals. The test data-set corresponds to the last six months of the whole time series (blue dotted line for GPP and red for ER). Predictions before Jan 2014 (gray dotted line) have not been considered for the error estimation as they have been used when training the ensemble algorithm.

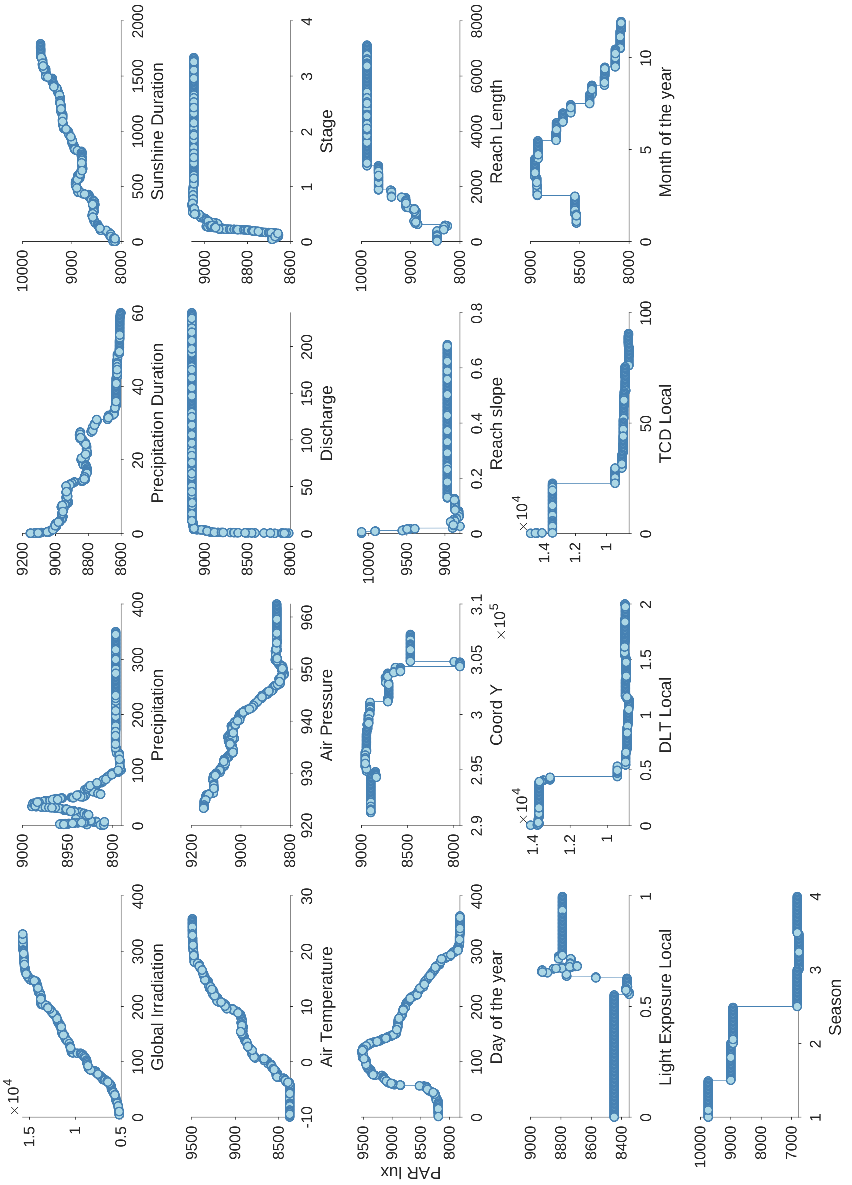

Figure S31: Partial dependence plots for the final PAR RF (Figure 2 of the main text). Each subplot shows the averaged (over the twelve trained forests) response variable predicted when all features except for that specified on the x label are kept constant and equal to their observed average.

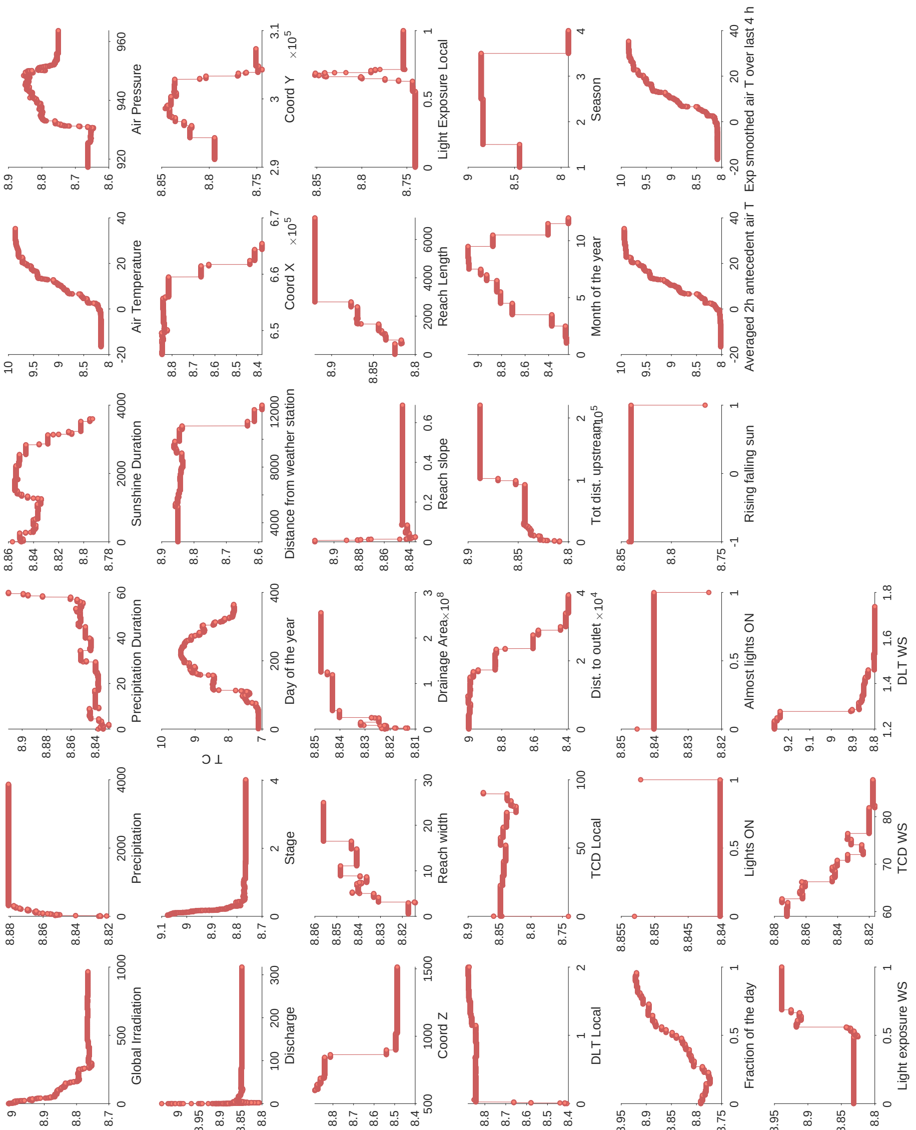

Figure S32: Partial dependence plots for the final T RF (Figure 2 of the main text). Each subplot shows the averaged (over the twelve trained forests) response variable predicted when all features except for that specified on the x label are kept constant and equal to their observed average.

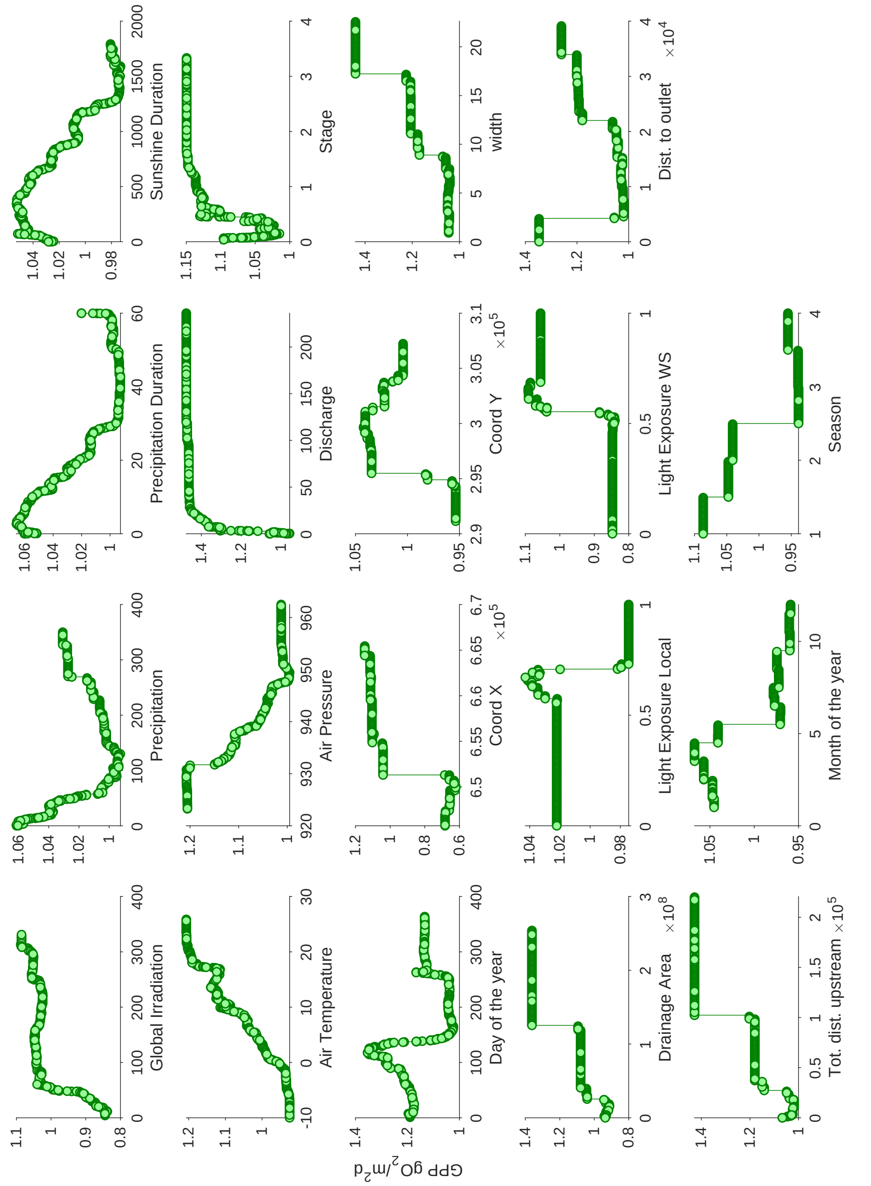

Figure S33: Partial dependence plots for the final GPP RF (Figure 3 of the main text). Each subplot shows the averaged (over the twelve trained forests) response variable predicted when all features except for that specified on the x label are kept constant and equal to their observed average.

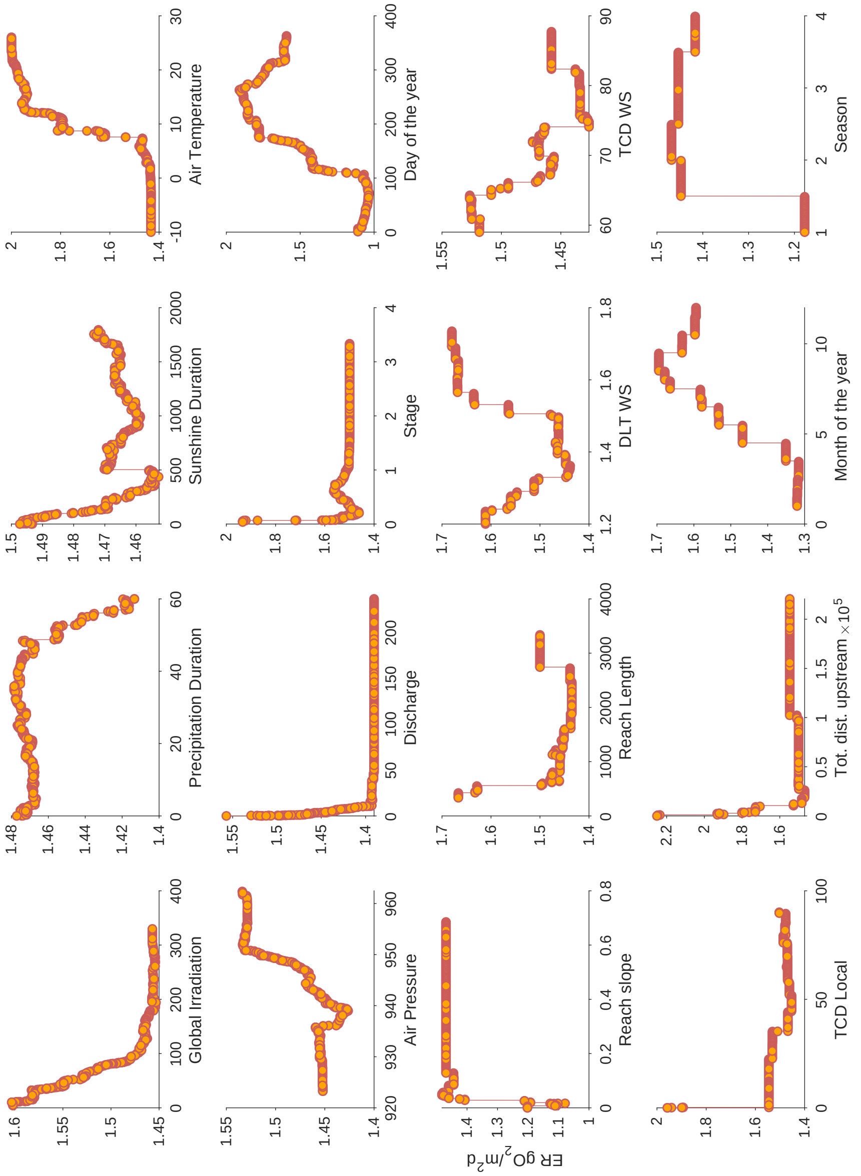

Figure S34: Partial dependence plots for the final ER RF (Figure 3 of the main text). Each subplot shows the averaged (over the twelve trained forests) response variable predicted when all features except for that specified on the x label are kept constant and equal to their observed average.

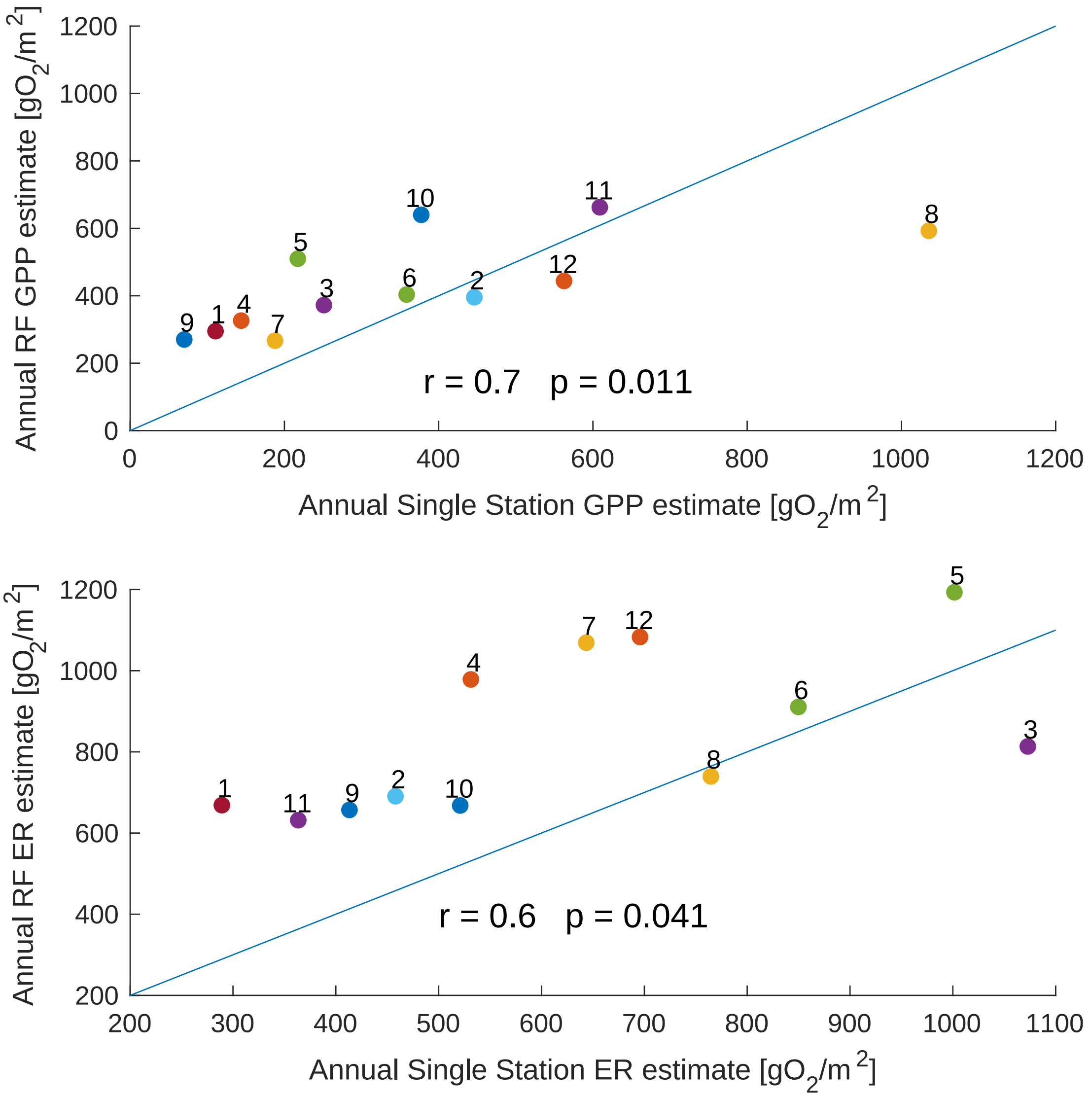

Figure S35: Cumulative areal GPP and ER estimated using the single station approach against modeled by means of the RF algorithm.

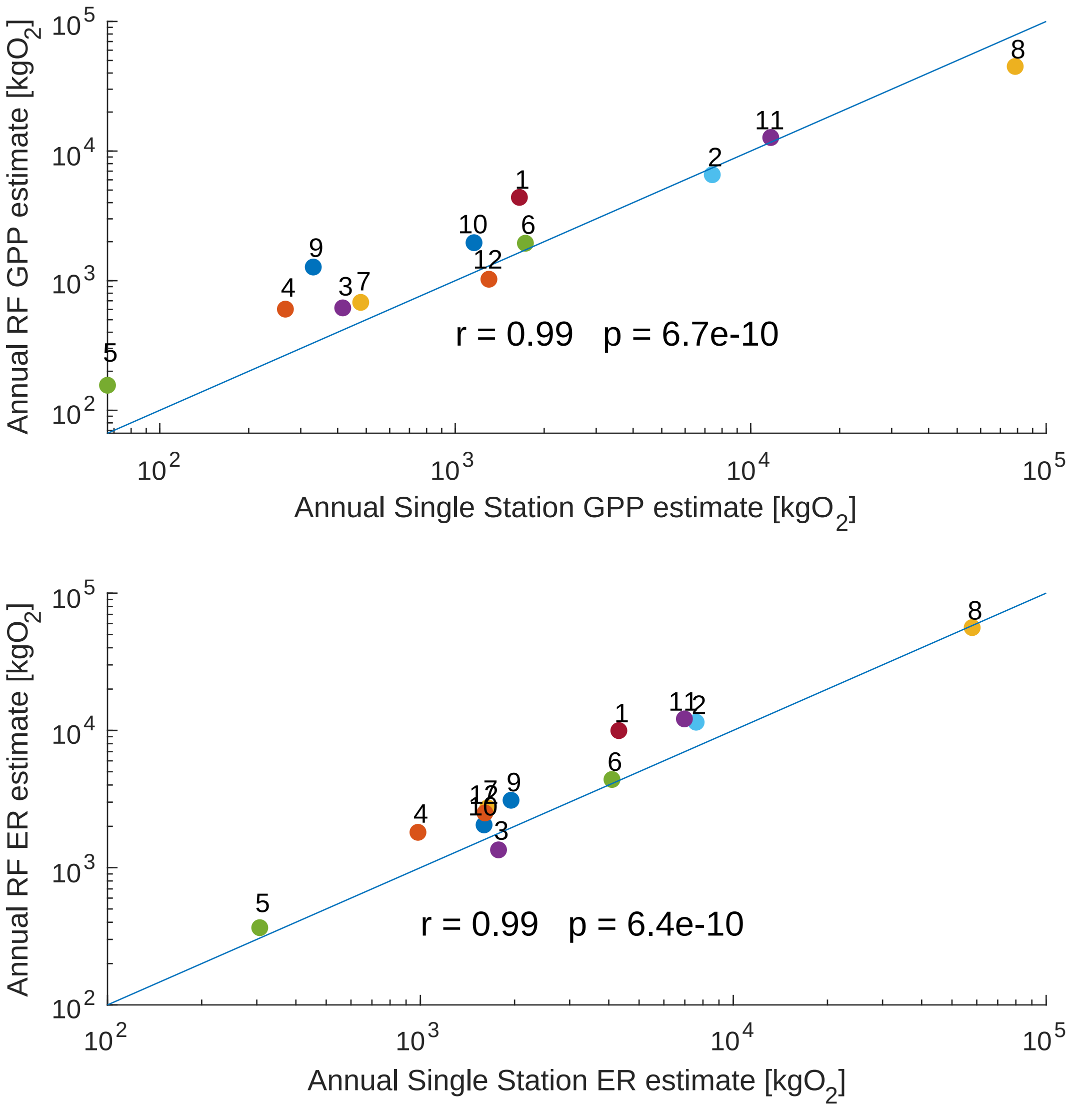

Figure S36: Cumulative GPP and ER mass estimated using the single station approach against modeled by means of the RF algorithm.
